# Cyclen Tetra‐Amide Ligands as a Privileged Class of Ligands for Studying Second‐Sphere Coordination Environments

**DOI:** 10.1002/chem.71136

**Published:** 2026-05-15

**Authors:** Andrea L. Batchev, Widana Kaushalya, Chashitha Padukka, Janya Subasinghe, Alice R. Walker, Matthew J. Allen

**Affiliations:** ^1^ Department of Chemistry Wayne State University Detroit Michigan USA

**Keywords:** hydrogen‐bond, MRI contrast agent, oxidation, privileged ligand, second‐sphere coordination

## Abstract

We propose that derivatives of cyclen tetra‐amides are a privileged class of ligands for large ions such as lanthanides that enable control over the second‐coordination sphere environment of discrete complexes. We demonstrate this concept first through highlighting examples of lanthanide cyclen tetra‐amide derivatives that provide control over three different second‐sphere environments. Control over the second‐coordination sphere is then leveraged to enable the first mechanistic and kinetic oxidation studies of a Eu^II^‐containing complex in solution. We observed oxidation rates that are dependent on the concentration of oxygen exposed to the Eu^II^ complex, with implications for quantifying hypoxia. Computational modeling of the inner‐ and second‐sphere coordination environments demonstrates that the protonation state of the phosphonate pendant arms of the Eu^II^ complex controls the access of O_2_ to the Eu^II^ innersphere via hydrogen bonding to second‐sphere water. Together, these findings provide a clear picture of the mechanism of the slow oxidation of the Eu^II^‐containing complex in solution and serve as a step toward the quantification of hypoxia and rational design of future Eu^II^‐containing complexes for imaging hypoxia in vivo. We expect that the molecular insights described here will inspire the study of other such privileged ligands for overcoming hurdles of rational design.

## Graphical abstract

Cyclen tetra‐amide derivatives provide a privileged ligand platform for controlling second‐coordination sphere structure. Hydrogen‐bond networks in the second‐coordination sphere are leveraged to stabilize a Eu^II^‐containing cyclen tetra‐amide derivative in physiologically relevant conditions by limiting the access of O_2_, establishing a design‐principle for redox‐active metals.

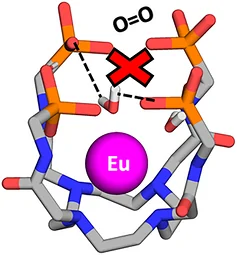

## 1 Introduction

Privileged molecules are used in nature and by scientists for their abilities to interact with a wide variety of partners or environments due to inherent structural and reactive properties [1, 2, 3, 4, 5]. In coordination chemistry, privileged ligands guide coordination chemistry toward useful outcomes by providing predictable and tunable control over the properties of metal complexes [6, 7, 8, 9, 10]. Understanding and controlling the coordination environments of metal complexes is an extremely powerful tool for molecular design that touches nearly all aspects of modern life. The first line of control in coordination chemistry is the inner coordination sphere in which atoms directly coordinate metals to form the core determinants of structure, stability, reactivity, and spectroscopic properties of coordination complexes. Inner‐sphere coordination chemistry is well established, but moving a layer away from the metal, the ability to rationally control environments becomes exponentially more challenging. The second coordination sphere is comprised of the molecular microenvironment that surrounds the inner sphere, in which noncovalent interactions are the dominant drivers. Where inner‐sphere coordination chemistry enables coarse tuning of chemical properties, control over the second sphere provides selectivity with respect to access of molecules to the inner sphere and over fine tuning of the properties of complexes [11, 12, 13]. Consequently, second‐sphere coordination chemistry has the potential to play important roles in the advancement of modern technologies. The majority of strategies to control second‐sphere coordination environments exist within large, complicated systems such as proteins and supramolecular adducts that require a complexity well beyond that of discrete metal complexes [13, 14, 15, 16]. We propose that tetra‐amide derivatives of cyclen, specifically derivatives of 1,4,7,10‐tetraazacyclododecane‐1,4,7,10‐tetraacetamide, are privileged ligands for control of the second coordination sphere environment of lanthanide ions. Here, we support this claim of privilege in three parts: first, we make the case for privilege through highlighting reported examples of tuning second‐sphere coordination chemistry with derivatives of this ligand. Next, we expand upon those examples by providing experimental evidence for control over the second sphere by studying a Eu^II^‐tetra‐amide complex, europium(II) 1,4,7,10‐tetra‐azacyclododecane‐1,4,7,10‐tetraacetamidomethylene phosphonic acid (Eu^II^ **1**). Finally, we demonstrate a method for computational modeling of the inner‐ and second‐coordination spheres of Eu^II^ **1** as a roadmap to studying second‐sphere environments of other derivatives of the privileged ligand.

## 2 Results and Discussion

**FIGURE 1:**
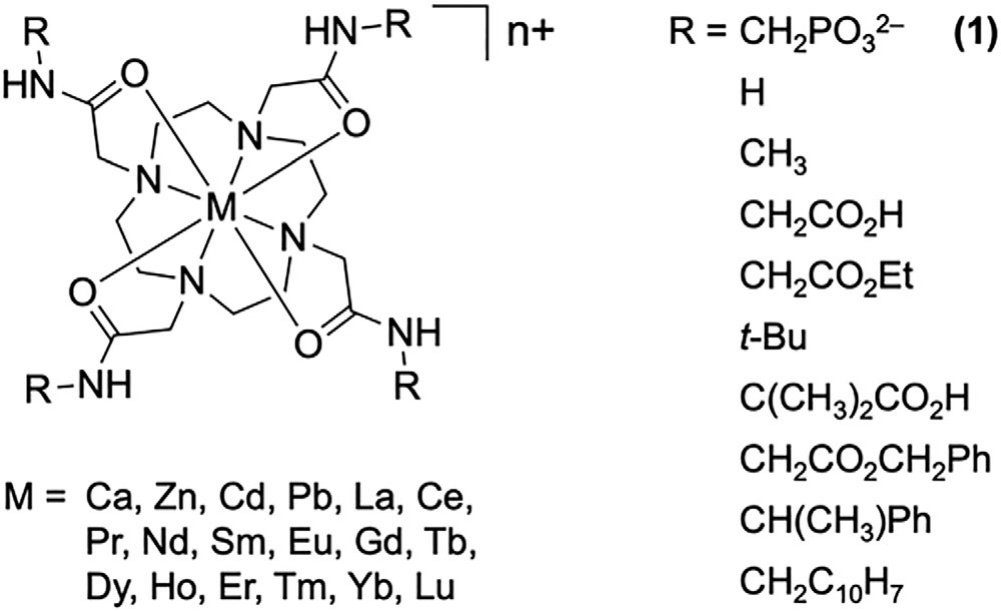
A selection of reported N‐substituted cyclen tetra‐amide derivatives [17, 18, 19, 20, 21, 22, 23, 24, 25, 26, 27, 28].

The tetra N‐substituted cyclen tetra‐amide (Figure 1) is a privileged ligand for the coordination of large metal ions like the lanthanides [17, 18, 19, 20, 21, 22, 23, 24, 25, 26, 27, 28]. Derivatives of cyclen tetra‐amides have a broad array of applications in catalysis [26], sensors and luminescence [17, 28, 29, 30], and medical imaging [20, 31, 32, 33, 34]. The tetra‐amides are an important feature on the cyclen‐ligand backbone that distinguishes cyclen tetra‐amides from other cyclen derivatives because they are extraordinarily useful for reliable control of the second coordination sphere. The four amide moieties on the pendant arms have significant effects on the water exchange of Ln‐cyclen tetra‐amides compared to other Ln‐cyclen derivatives such as tetracarboxylates and tetraphosphonates. This effect can be attributed to strong Ln–OH_2_ bonds caused by electron‐withdrawing effects of the amides and a stabilizing effect of hydrogen‐bonding networks created by the amides in the second sphere [18, 35, 36]. Cyclen tetra‐amide derivatives are also easily modifiable, and modifications to the pendant arms offer an avenue toward rational control of the second coordination sphere. Here, we make the case that cyclen tetra‐amides are privileged ligands for control of the second coordination sphere by providing three examples that demonstrate control over different aspects of the second‐sphere environment: water exchange, proton exchange, and the access of O_2_ to the inner sphere.

**FIGURE 2:**
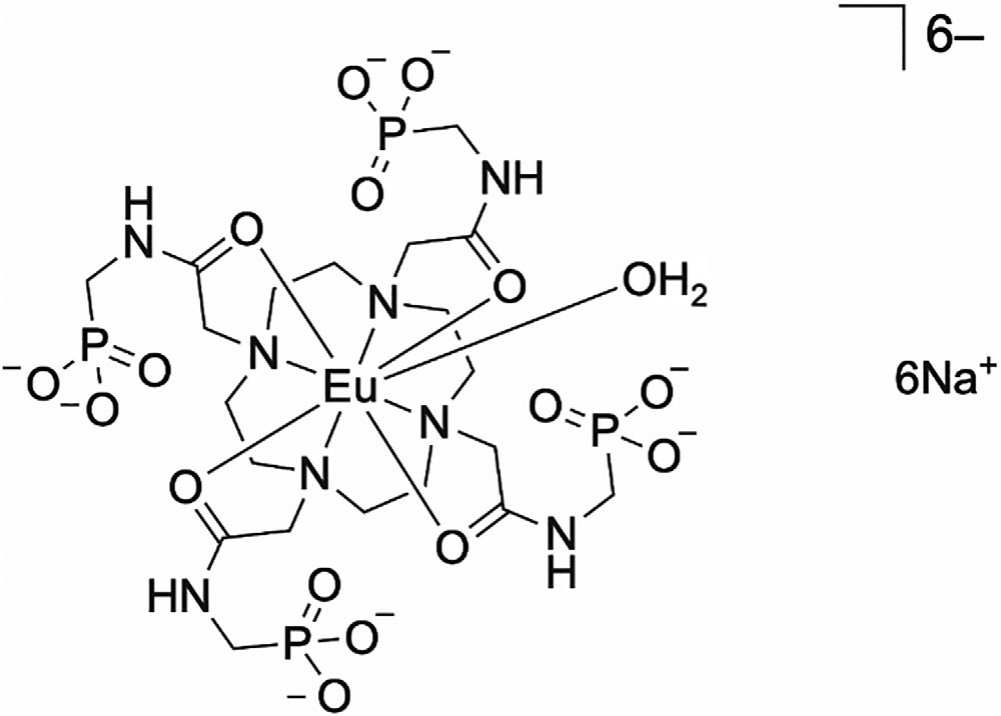
Eu^II^ **1** with fully deprotonated phosphonate pendant arms [Eu^II^ **1**]^6–^. Correction updated on 15th June 2026, Figure 2 was updated in this version.

Control over water exchange was achieved using a cyclen tetra‐amide derivative containing four *p*‐trifluoromethylbenzyl groups on the amide pendant arms of a redox‐ and temperature‐sensitive Eu^II^‐containing contrast agent for MRI [37]. At low temperatures, Π‐stacking interactions and the hydrophobicity of the trifluoromethyl groups force the pendant arms into a pseudo‐axial position, forming a cage around a coordinated water molecule that minimizes inner‐sphere contributions to relaxivity by blocking water exchange from the inner to the second sphere. By increasing temperature, thermal energy competes with these interactions, enabling coordinated water to exchange through the second sphere to increase relaxivity. In the next example highlighted here, modifications to the amide‐containing pendant arms afford control over the second‐sphere environment in two aspects through the formation of hydrogen‐bond networks in complexes of 1,4,7,10‐tetra‐azacyclododecane‐1,4,7,10‐tetraacetamidomethylene phosphonic acid (ligand **1**, Figure 2). In ligand **1**, hydrogen‐bonding networks created by the four phosphonate‐containing pendant arms modulate relaxivity as a function of pH when complexed with Gd^III^ [33, 38, 39]. Partial protonation of the phosphonate arms results in prototropic exchange of a coordinated inner‐sphere water molecule with second‐sphere protons [38]. The prototropic exchange is catalyzed as a function of the protonation state of the phosphonates. Additionally, chelates of **1** containing Eu^II^ exhibit a pH‐dependent resistance to oxidation by O_2_, in which the resistance of Eu^II^ **1** to oxidation after exposure to O_2_ was found to increase as pH increased over a range of pH = 7–10 (Table 1) [40]. It was theorized that when the pH is greater than the p*K_a_* of the phosphonate arms, a cage‐like structure forms between the hydrogen bonds of deprotonated phosphonate arms and an inner‐sphere water molecule, thereby hindering the access of O_2_ and increasing the persistence of Eu^II^ in oxygenated solutions.

**TABLE 1:** pH‐dependant oxidation of Eu^II^1 after exposure to air [40].

pH	t_1/2_ (min)
7	6.4 ± 0.4
8	8.0 ± 0.4
9	8.8 ± 0.4
10	12.4 ± 0.1

The oxygen resistance of Eu^II^‐containing complexes is not observed for other phosphonate‐ or amide‐containing ligands that exhibit second‐sphere contributions to relaxivity but lack the macrocyclic tetra‐amide backbone. For example, Eu^II^‐1,4,7,10‐tetraazacyclododecane‐1,4,7,10‐tetra(methylenephosphonate) (Eu^II^‐DOTP) contains four terminal phosphonate groups but lacks the tetra‐amide scaffold and does not exhibit resistance to oxidation by O_2_ [36, 40]. Similarly, Eu^II^ complexes of diethylenetriaminepentaacetic acid (DTPA) display second‐sphere relaxivity but show lower oxygen stability than Eu^II^_(aq)_ and water exchange rates nearly three orders of magnitude faster than the corresponding Gd^III^ complex [41]. Together, these counterexamples demonstrate that although phosphonate‐ and amide‐containing ligands can contribute second‐sphere effects, the cyclen tetra‐amide scaffold uniquely enables precise control over the second coordination sphere.

Therefore, ligand **1** provides an opportunity to explore the use of a privileged ligand for second‐sphere control. We chose to use Eu^II^ **1** as our model system because the slow oxidation of Eu^II^ **1** enables an investigation of the mechanism and kinetics of Eu^II^ oxidation in oxygenated solution, which has impactful implications in medical diagnostics for hypoxia sensing. Hypoxia results when oxygen supply is inadequate for tissue oxygen needs. This imbalance leads to a host of physiologic [42, 43, 44], metabolic [42, 43, 44], and immunologic changes [42, 45]. In reducing environments, Eu^II^ enhances contrast by increasing the relaxivity of nearby water protons, similarly to isoelectronic Gd^III^ [46, 47]; however, Eu^II^ is oxidized to Eu^III^ by molecular oxygen, and Eu^III^ does not enhance *T*_1_‐weighted contrast in MRI. The electronic differences between Eu^II^ and Eu^III^ enable their facile detection for imaging hypoxia and other oxygen‐deficient environments [37, 48, 49, 50], although rapid and irreversible oxidation of Eu^II^ to Eu^III^ in normoxic environments provides an obstacle to the use of Eu^II^‐containing complexes in a wider range of hypoxic environments.

Eu^II^ **1** was the first Eu^II^‐containing complex reported to enhance contrast in MRI after systemic delivery [40], and although the use of Eu^II^ **1** in systemic delivery marks a great step toward the use of Eu^II^‐containing complexes to practically image hypoxia, Eu^II^‐containing complexes need to persist long enough in oxygenated environments to enable imaging of distant sites after systemic injection. The mechanisms underlying the oxidation of Eu^II^ **1** must be further understood before undertaking the design of long‐lasting Eu^II^‐containing complexes in oxygenated environments or before quantification of hypoxia can occur. We hypothesized that exposure of Eu^II^ **1** to different oxygenated environments would lead to differences in Eu^II^ **1** oxidation rate visualizable by UV–visible spectroscopy. Additionally, the inner‐ and second‐sphere coordination environments of Eu^II^ **1** were modeled in multiple protonation states using classic molecular dynamic simulations with explicit solvent to investigate the effects of protonation on Eu^II^ **1** conformation and hydrogen‐bonding interactions in the absence and presence of O_2_. Here, we present mechanistic studies of the oxidation of Eu^II^ **1** in solutions containing different amounts of O_2_ and in silico as a step toward the quantification of hypoxia.

**FIGURE 3:**
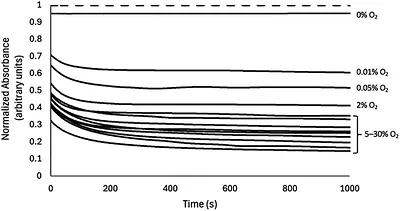
UV–visible spectroscopy signal decay data monitored at λ = 420 nm for Eu^II^ **1** after sparging with gas (50 mL). The dashed line signifies time‐course data acquired without sparging. From top solid line and ending at the bottom solid line, the gas sparged into the cuvette was 0%, 0.01%, 2%, 5%, 8%, 10%, 12%, 15%, 18%, 20.9%, 23.4%, and 30% O_2_ in N_2_.

The ability of a Eu^II^‐containing complex to persist in oxygenated environments opens many doors toward the use of Eu^II^ for visualizing hypoxia in practical contexts. A first step toward the quantification of hypoxia using Eu^II^ **1** is understanding how Eu^II^ **1** behaves in solution in the presence of different oxygen concentrations. We hypothesized that Eu^II^ **1** would oxidize at different rates depending on the amount of oxygen that was exposed to Eu^II^ **1** because our previous report showed that Eu^II^ **1** persists even after exposure to approximately 60 equivalents of O_2_ [40]. Similar to the difference in magnetic properties between Eu^II^ **1** and Eu^III^ **1** that enables the MR imaging of hypoxia, Eu^II^ **1** and Eu^III^ **1** also have different absorption spectra; therefore, oxidation of Eu^II^ **1** to Eu^III^ **1** can be monitored through UV–visible spectroscopy. To test this hypothesis, we exposed solutions of Eu^II^ **1** (3.5 mM, 2 mL) to gas (50 mL) containing different concentrations of O_2_ (between 0% and 30% O_2_ in N_2_) and observed the changes in UV–visible absorbance spectra (λ = 420 nm) over time (3600 s). We selected a range of gases that are physiologically relevant in hypoxic and normoxic conditions, as well as O_2_ concentrations greater than those found in atmospheric air, to gain a more thorough understanding of Eu^II^ **1** oxidation in solution. The wavelength 420 nm was selected because Eu^II^ **1**, and not Eu^III^ **1** nor the Eu aqua ion, absorbs at 420 nm light. The data from each experiment were normalized to the initial absorbances of each sample before exposure to O_2_, and each experiment was performed in triplicate. The means of the triplicates were overlaid for comparison (Figures 3 and S1–S13). The initial point on each curve represented the largest absorption. Each curve decayed after the sparge that took place before the start of spectroscopy data collection until a plateau was reached, except for the two control experiments that did not change significantly in absorbance across the time course. Generally, the initial absorbances of the averages of each curve show decreases along the y‐intercept with increasing concentration of O_2_. The curves corresponding to 0 and 30% O_2_ have the greatest and least initial absorbances, respectively, and the only curve that does not follow the trend of decreasing mean normalized initial absorbance with increasing O_2_ concentration was 23.4%, which showed an initial absorbance close to the mean of 18%. The curves between 5 and 23.4% O_2_ also show crossovers that occur primarily in the first 200 s. Conversely, the initial absorbances for the curves corresponding to 0%–2% O_2_ are particularly well delineated. In addition to lower initial absorbances, the greater the concentrations of O_2_ sparged into cuvettes, resulted in smaller he final absorbances and the longer the times until initial plateaus in decay were reached. Additionally, there were decreases in absorbance after the initial plateau, usually occurring after 2000 s. Two control experiments were performed to test the experimental setup. The first control experiment was performed without sparging gas into solutions of Eu^II^ **1** (Figure 3, dashed line). In this control experiment, there was no change in absorbance over time, indicating that there was no oxidation of Eu^II^ **1** to Eu^III^ **1** in the absence of added gas. The second control experiment was performed by sparging N_2_ gas into the cuvettes containing solutions of Eu^II^ **1** (Figure 3, first solid line below the dashed line). Although N_2_ does not oxidize Eu^II^ **1**, there was a normalized average absorbance of 95% at the start of the time course, indicating that there was a small but measurable amount of O_2_ leakage from the atmosphere into the system during sparging or from trace amounts of O_2_ in the N_2_ gas. Because most of the decay of absorbance occurred before the start of the collection of UV–visible spectroscopy data, most of the oxidation that occurred during these experiments occurred during the sparge, when oxygen was directly introduced to the aqueous solutions of Eu^II^ **1**. These results are consistent with the oxidation of Eu^II^ **1** previously reported after sparging with atmospheric air [40]. In all experiments, incomplete oxidation of Eu^II^ **1** occurred, including experiments with greater than atmospheric concentrations of O_2_. Eu^II^ **1** is persistent at oxygen levels even greater than that of atmospheric oxygen, and the rate of oxidation of Eu^II^ **1** at oxygen concentrations between 0%–2% is well distinguishable. The ability to distinguish concentrations of O_2_ between 0% and 2% is particularly important because hypoxia is generally recognized to occur at pO_2_ levels less than or equal to 15 mmHg (≤2% O_2_) [51]. Together, these results signify the potential for the quantification of Eu^II^ **1** oxidation based on O_2_ concentration and time.

**FIGURE 4:**
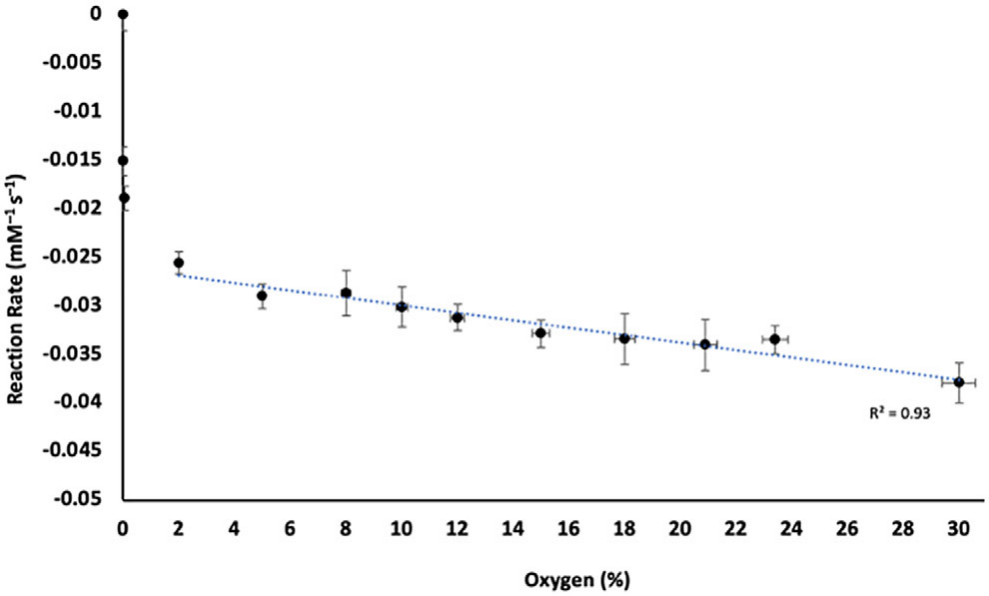
Reaction rate of Eu^II^ **1** after introduction of varying concentrations of O_2_ gas. Vertical error bars show the standard error of the mean of the reaction rate measurements, and horizontal error bars depict the error associated with the concentration of O_2_ in the independently prepared calibration gases.

We also wanted to investigate whether there were any trends in the rates of reaction of Eu^II^ **1** and O_2_. We initially hypothesized that the reaction rates of Eu^II^ **1** with O_2_ would be dependent on the concentration of O_2_ that was bubbled into the cuvettes because the reaction of Eu^II^ **1** with different concentrations of O_2_ gas showed replicable initial absorbances depending on the concentration of O_2_. To determine the oxidation rate of Eu^II^ **1**, the amount of Eu^II^ **1** that oxidized to Eu^III^ **1** at the start of data collection after sparging was converted to concentration (mM) using the Beer–Lambert law. The change in calculated concentration was divided by the time it took from the start of sparging to the time of first data point collection. The oxidation rates and corresponding errors were calculated and plotted against the concentration of O_2_ that was bubbled through the solutions of Eu^II^ **1** (Figure 4). There appear to be two different regions of reaction‐rate trends of Eu^II^ **1** oxidation depending on the concentration of O_2_. The first trend occurs at small concentrations of O_2_ (≤0.05%), and the second trend occurs at concentrations of O_2_ that are  ≥2%. The trend at small concentrations of O_2_ shows an intense dependence on the oxidation rate of Eu^II^ on the concentration of O_2_. The second trend shows a less intense dependence of oxidation rate on the concentration of O_2_. To explain these trends, we looked at the stoichiometry of O_2_ bubbled into the system compared to Eu^II^ **1** in solution. Because 50 mL of O_2_ was bubbled into each trial, we calculated the total moles of O_2_ introduced to the solution as the upper limit of equivalents able to react with Eu^II^ **1**. The upper limit equivalents of O_2_ ranged from 100 to 0.03 for 30% and 0.01% O_2_, respectively (Table 2). However, the cuvettes have finite volumes, and oxygen has a solubility of approximately 258 mM in water at standard temperature and pressure [52], resulting in most of the O_2_ entering the headspace of the cuvette or escaping through the purge needle during sparging. The lower limit of equivalents of O_2_ was calculated as the moles of O_2_ soluble in the volume of solution in the cuvettes, combined with the moles of O_2_ occupying the headspace of the cuvette. The lower limits of equivalents of O_2_ were between 9.6 and 0.001 for 30 and 0.01%, respectively (Table 2). It is unlikely that all molecules of O_2_ bubbled through the solutions contact Eu^II^ **1** or that none of the molecules of O_2_ interact with Eu^II^ **1** while bubbling; therefore, we reasoned that the actual number of molecules of O_2_ available to interact with Eu^II^ **1** in solution must fall between these two limits. With these limits in mind, we calculated the approximate rate order for O_2_ using steady‐state approximations for 0.01% and 0.05% O_2_ (see Equations S1–S6) because Eu^II^ **1** was in excess to O_2_ by at least 100‐fold at both the upper and lower limits of O_2_ stoichiometry. We found the rate order of O_2_ to be 0.3 and 0.2 for the lower and upper limits, respectively, at concentrations of 0.01% and 0.05% O_2_. Additionally, we made steady‐state approximations to determine the reaction order of Eu^II^ **1** (see Equations S7–S10) at concentrations in excess of atmospheric oxygen (23.4% and 30% O_2_), where O_2_ is in excess of Eu^II^ **1** by at least 100‐fold at the upper limit. At the upper limit, the reaction order of Eu^II^ **1** in 23.4% and 30% O_2_ was 1.2. The stoichiometry at the lower limit was not appropriate for steady‐state approximations to be valid. The noninteger reaction orders calculated for Eu^II^ **1** and O_2_ are not surprising because of the complexity of the system: ligand **1** contains four phosphonate groups that each contain two protonatable oxygens. Ligand **1** was previously studied as a pH‐responsive contrast agent when metallated with Gd^III^ [33, 38, 39]. At pH 7.5, it was reported that Gd^III^ **1** exists in three different protonation states [38]: a diprotonated species [Gd**1**H_2_]^3−^ (∼2%), a monoprotonated species [Gd**1**H]^4−^ (∼33%), and a fully deprotonated species [Gd**1**]^5−^ (∼65%). The pH sensitivity of Gd^III^ **1** was attributed to differences in the proton lability of an inner‐sphere water molecule caused by hydrogen bonding of the phosphonate groups. It is possible that similar protonation states influence access of O_2_ to Eu^II^ in Eu^II^ **1**. Additionally, O_2_^−^ is a possible byproduct of the oxidation of Eu^II^ by O_2_ [53]. The production of O_2_^−^ after oxidation of Eu^II^ **1** could lead to the oxidation of other complexes of Eu^II^ **1**, thereby complicating the determination of the reaction kinetics of Eu^II^ **1** and O_2_. The noninteger reaction orders found using steady‐state approximations for O_2_ and Eu^II^ **1** indicate that a complex oxidation reaction occurs between O_2_ and Eu^II^ **1** that might include interactions between Eu^II^ **1** and byproducts of its oxidation by O_2_ and likely includes multiple protonation states of Eu^II^ **1**.

**TABLE 2:** Upper and lower limit equivalents of O_2_ relative to Eu^II^ **1** and reaction rates of oxidation of Eu^II^ **1**.

O_2_ (%)	Upper limit (equiv.)	Lower limit (equiv.)	Reaction rate (mM^−1^ s^−1^)
30	100	9.6	−0.038 ± 0.002
23.40	75	2.3	−0.033 ± 0.001
21	66	2.1	−0.034 ± 0.003
18	57	1.8	−0.033 ± 0.003
15	48	1.5	−0.033 ± 0.001
12	38	1.2	−0.031 ± 0.001
10	32	1.0	−0.030 ± 0.002
8	30	0.8	−0.029 ± 0.002
5	20	0.6	−0.029 ± 0.001
2	10	0.3	−0.026 ± 0.001
0.05	0.2	0.08	−0.019 ± 0.001
0.01	0.03	0.001	−0.015 ± 0.001
0	0	0	Not applicable

The reaction rates were adjusted to rates normalized to the control of 0% O_2_.

**FIGURE 5:**
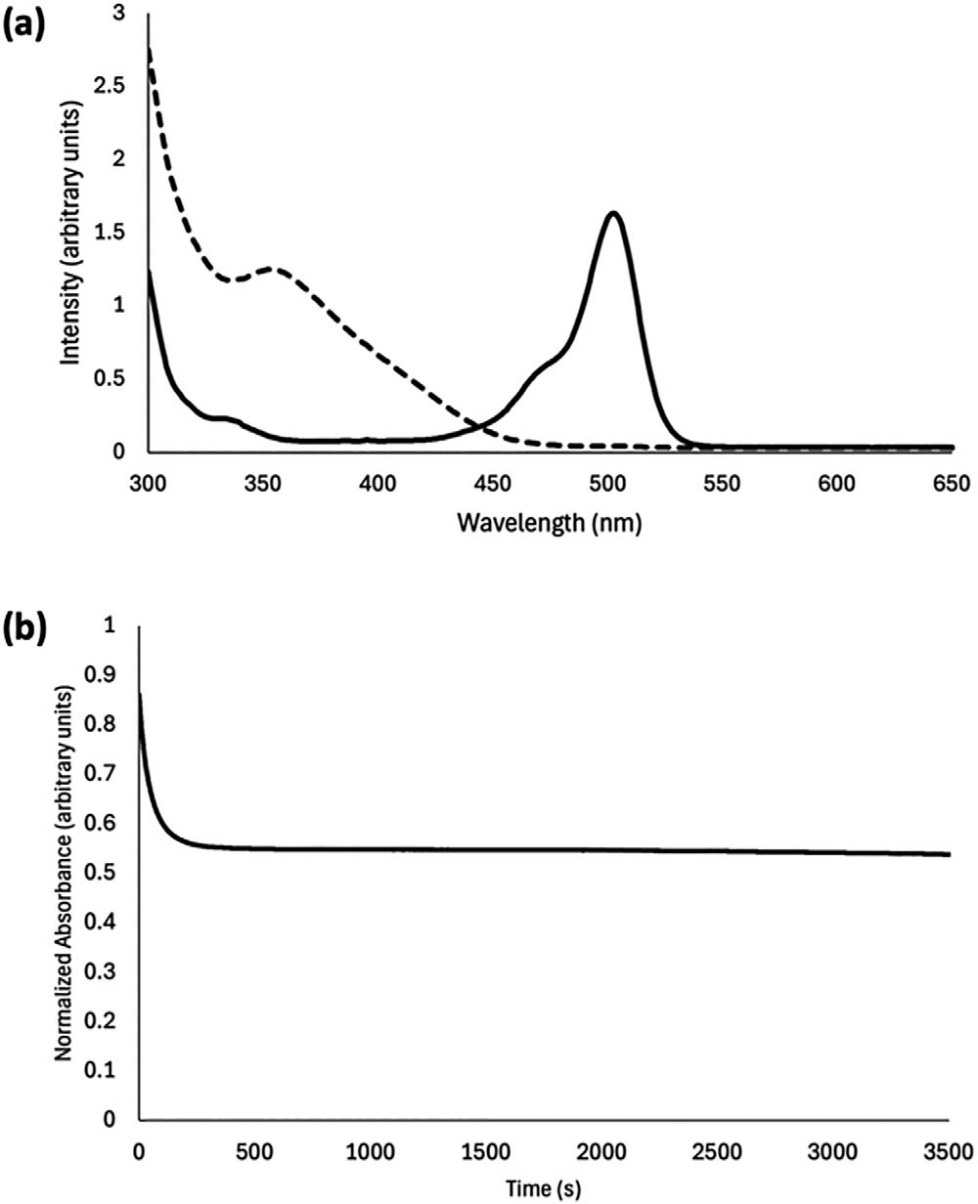
(a) UV–visible spectra of a mixture of Eu^II^ **1** and 7,10‐dichlorofluoresceine before (**‐ ‐**) and after (—) sparging with atmospheric gas. (b) Representative UV–visible time course spectrum of Eu^II^ **1** after the addition of potassium superoxide dissolved in degassed water.

Because superoxide is a potential by‐product of Eu^II^ oxidation by O_2_ that can potentially oxidize Eu^II^, we wanted to test if the possible production of superoxide might lead to some of the oxidation of Eu^II^ **1** after the initial sparging of O_2_, because there is some decay in absorbance intensity after starting the absorbance time course. To this end, we performed experiments with 2′,7′‐dihydrochlorofluorescein that is widely used in superoxide and reactive‐oxygen species assays because it is hydrophilic and its oxidation by reactive oxygen species yields fluorescent 2′,7′‐dichlorofluorescein that has a maximum absorbance around 500 nm [54, 55]. To detect the production of O_2_^−^ after the oxidation of Eu^II^ **1** by O_2_, the UV–visible spectra of samples of Eu^II^ **1** containing 2′,7′‐dichlorofluorescein were acquired before and after sparging with air (Figure 5a). Before sparging, samples containing Eu^II^ **1** and 2′,7′‐dichlorfluorescein were pale yellow, but several minutes after sparging, the samples turned bright green. The absorbance at 500 nm drastically increased after sparging samples containing Eu^II^ **1** and 2′,7′‐dichlorfluorescein with air (Figure 5a). These results indicate that O_2_^−^ is produced after Eu^II^ **1** oxidizes to Eu^III^ **1** after exposure to O_2_. This observation is consistent with previous studies that demonstrate the production of O_2_^−^ after oxidation of Eu^II^‐containing complexes O_2_ [53], and it makes sense intuitively because O_2_^−^ is the by‐product of a one‐electron transfer to O_2_.

Superoxide anions are radical species that are unstable and act as both reductants and oxidants. Although O_2_^–^ is considered a reactive oxygen species, it is a selective oxidant that plays an important role in cell signaling and immune defense pathways [56]. To see if O_2_^−^ oxidizes Eu^II^ **1**, UV–visible decay time courses were repeated with the addition of an aqueous solution of KO_2_ instead of O_2_ gas (Figure 5b). We observed a fast but incomplete signal decay that plateaued despite the addition of one equivalent of O_2_^−^ and Eu^II^ **1**. Notably, the decay of signal associated with Eu^II^ **1** after addition of O_2_^−^ led to a plateau that did not further decay up to one hour, unlike the time‐course experiments that added oxygen. These results indicate that O_2_^−^ reacts with Eu^II^ **1**. The oxidation of Eu^II^ **1** by O_2_^−^ is interesting because there are reports of isolatable lanthanide complexes with O_2_^−^ as a bidentate ligand [57, 58]; however, Eu^II^ **1** only has one coordination site available for coordination with O_2_^−^. The reaction between O_2_^−^ and Eu^II^ **1** likely contributes to the noninteger reaction orders that we measured with O_2_, but it is not responsible for the decay in signal observed beyond 2,000 s after the addition of oxygen that does not fully oxidize Eu^II^ **1** due to the speed of the sharper decrease in absorbance followed by a plateau that persists for the entire time course compared to the sparged time course experiments (Figures S1–S13).

**FIGURE 6:**
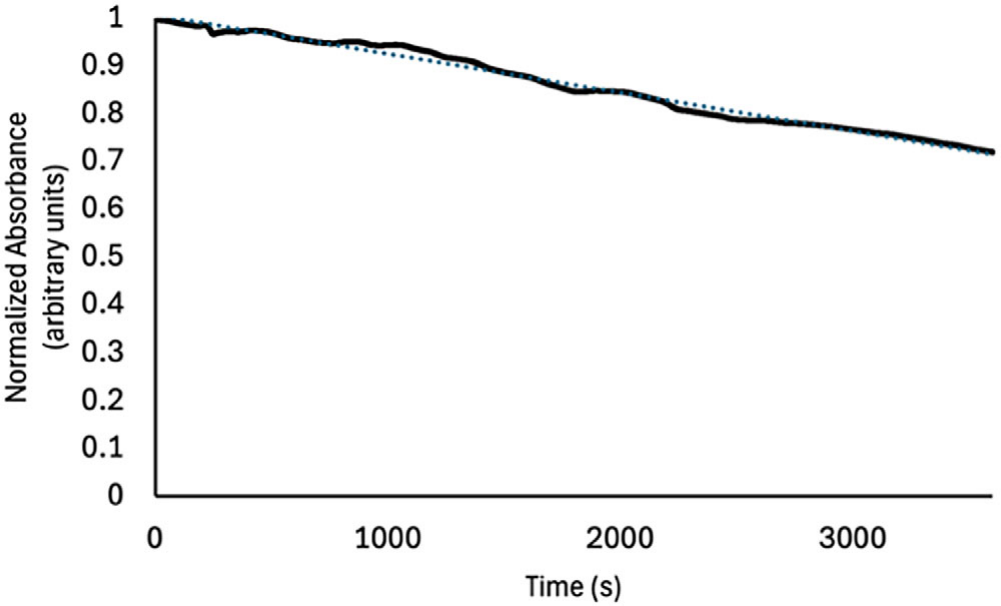
Representative UV–visible spectrum of Eu^II^ **1** after sparging air into the headspace of the cuvette. The solid line depicts the change in absorbance at λ = 420 nm over time, and the dotted line depicts the line of best fit.

To explain these observations, we hypothesized that oxygen from the headspace of the cuvettes diffuses into solution over time and, subsequently, reacts with Eu^II^ **1**, causing oxidation and signal decay after the initial plateau. To explore the contributions of oxygen diffusion from the headspace to solutions of Eu^II^ **1**, we repeated UV–visible time‐course studies but introduced oxygen only to the headspace of the cuvettes instead of directly into the solution. The signal decay observed was different from those of the time courses that sparged oxygen directly into the solution and from the addition of KO_2_. Instead of a sharp signal decay that plateaued, a line with a relatively gentle slope was observed (Figure 6), and a line of best fit showed good linear agreement (R^2^ = 0.99). These results are due to the diffusion of oxygen from the headspace into the solution containing Eu^II^ **1**. The slow and incomplete oxidation of Eu^II^ **1** after the addition of oxygen into the headspace is consistent with the low solubility of O_2_ in water at room temperature (approximately 0.5 micromoles in 2 mL) [52] and is the likely cause of the slow decay after the initial plateau observed in the gas bubbling experiments with different concentrations of O_2_ (Figures S1–S13).

## 3 Computational Modeling

**FIGURE 7:**
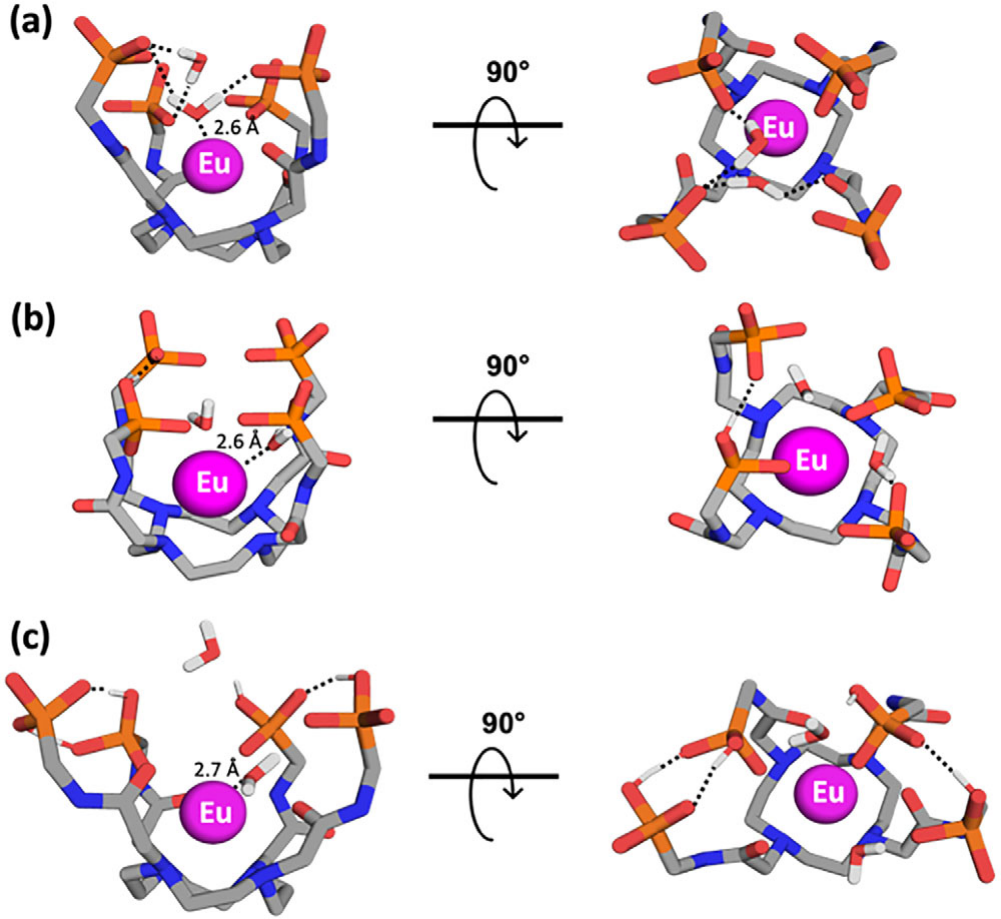
Representative geometries from classical molecular‐dynamics simulations of (a) [Eu^II^ **1**]^6−^, (b) [Eu^II^ **1**H]^5−^, and (c) [Eu^II^ **1**H_4_]^2−^ in water. The distances between Eu^II^ and the closest water molecules are shown in Å, with key hydrogen bonding between phosphonate arms and between phosphonate arms and water represented by dashed lines. Eu^II^ ions are shown as pink spheres, and water and ligands are shown as stick models.

To support our proposed explanation of the increased persistence to O_2_ by Eu^II^ **1** compared to other Eu^II^‐containing complexes in solution and to observe the inner‐ and second‐sphere coordination environments of Eu^II^ **1** and to support the claim of cyclen tetra‐amides being privileged ligands, we employed molecular dynamics simulations of Eu^II^ **1** in different protonated states in water. We simulated the fully deprotonated and monoprotonated states of Eu^II^ **1** because at pH 7.5, the fully deprotonated [Eu^II^ **1**]^6−^, Figure 7a) and monoprotonated [Eu^II^ **1**H]^5−^, Figure 7b) species are likely the majority species in solution based on the assumption that speciation is similar to that of the gadolinium complex [38]. The tetraprotonated [Eu^II^ **1**H_4_]^2−^, Figure 7c) species was also modeled for comparison because although it is likely not present in solution at pH 7.5. Rather, it should be the major species at pH 5 [38], and it should be the most susceptible species to oxidation by O_2_ based on our hypothesis. To make computational models, geometry optimization calculations of Eu^II^ **1** were performed in the gas phase and then in solution state using implicit water. The geometry‐optimized structures of Eu^II^ **1** in the solution state were used for molecular dynamics simulations with explicit water molecules. Classical molecular dynamics simulations of Eu^II^ **1** in solution revealed that protonation level governed the conformations of Eu^II^ **1** that resulted in different behaviors of the inner‐ and second‐sphere water molecules, where inner‐sphere water is directly coordinated to Eu in the model, and second‐sphere is hydrogen bound to the phosphonates within a pseudo‐cage formed by the phosphonates and Eu. Second‐sphere water molecules in the simulations formed hydrogen bonds with the phosphonate arms to varying degrees. We observed that with [Eu^II^ **1**]^6−^, the second‐sphere water molecule formed hydrogen bonds with the phosphonate arms throughout most of the simulation, drawing the arms together in a cage‐like formation and causing [Eu^II^ **1**]^6−^ to adopt a stable, compact formation (Figure 7a). The inner‐sphere water interchanges with the second‐sphere water molecule slowly (two times in 100 ns) without dissociating, further indicating the cage‐like formation in the closed form (Figure S15). In [Eu^II^ **1**H]^5−^, the monoprotonated phosphonate formed hydrogen bonds with a deprotonated phosphonate arm throughout most of the simulation and showed less interaction with second‐sphere waters than [Eu^II^ **1**]^6−^ (Figure 7b). The asymmetric hydrogen bonds between only two of the phosphonate arms rendered [Eu^II^ **1**H]^5−^ in an asymmetrically closed state. The partially open [Eu^II^ **1**H]^5−^ complex showed a longer overall distance for the inner‐ and second‐sphere waters (+0.05 Å) relative to [Eu^II^ **1**]^6−^, and a slightly more rapid exchange (four times in 100 ns) without dissociating (Figure S16). During molecular‐dynamics simulations of [Eu^II^ **1**H_4_]^2−^, transient hydrogen bonds formed between two pairs of phosphonate arms through approximately 20% of the simulation, opening the space around the europium ion for water exchange with bulk water (Figure 7c). The distance between Eu^II^ and the inner‐sphere water molecules in the molecular dynamics simulations of [Eu^II^ **1**]^6−^ and [Eu^II^ **1**H]^5−^ was each 2.6–2.7 Å, and the distance for [Eu^II^ **1**H_4_]^2−^ was 2.62–2.7 Å. The distances of the inner‐sphere water molecules for both fully and partially deprotonated Eu^II^ **1** complexes are consistent with distances between Eu^II^ and inner‐sphere water molecules observed in other Eu^II^ cyclen tetra‐amide complexes [37, 59]. However, in the more open [Eu^II^ **1**H_4_]^2−^ state, the second‐sphere water molecule experienced more exchange with outer‐sphere water and had a longer overall distance (∼5 Å) than in [Eu^II^ **1**]^6−^ or [Eu^II^ **1**H]^5−^. In [Eu^II^ **1**H_4_]^2−^ the second‐sphere water exchanged with bulk water rapidly (60 times in 100 ns) (Figure S17), but the inner‐sphere water molecule remained at a stable distance of around 2.68 Å over the course of the simulation (Figure S18). Classical molecular dynamics simulations were also performed with the addition of a molecule of O_2_ over the course of 1,000 ns to observe how the presence of hydrogen bonds affects access of O_2_ to different conformations of Eu^II^ **1** ([Eu^II^ **1**]^6−^, [Eu^II^ **1**H]^5−^, and [Eu^II^ **1**H_4_]^2−^) (Figures S19 and S20). In all conformations, water molecules preferentially occupied the Eu^II^ **1** inner sphere over O_2_ (Figure S20). Together, these simulations support our hypothesis that a cage‐like structure formed by the partially protonated phosphonate arms of Eu^II^ **1** helps slow the oxidation of Eu^II^ **1**. The openness of the complex and rate of interchange between an inner‐ and second‐sphere water molecule depend on the formation of hydrogen bonds between the phosphonate arms themselves or between the phosphonate arms and a second‐sphere water molecule (Figures S15–S18). We did not perform simulations long enough to observe water exchange between the inner‐sphere water molecules and bulk water. The differences in interchange between the inner‐sphere and second‐sphere water molecules that we observed in the classical molecular‐dynamics simulations of Eu^II^ **1** are consistent with the pH‐dependent relaxivity of Gd^III^ **1** [33, 38, 39]. Additionally, these observations are consistent with previous findings that Eu^II^ **1** oxidizes faster at pH 7 than at pH 10 [40].

## 4 Conclusion

We make the case that cyclen tetra‐amide is a privileged ligand with regard to control over second‐sphere coordination environments. The cyclen tetra‐amide scaffold exhibits slow‐water exchange due to the rigid macrocyclic framework that stabilizes bound water molecules through hydrogen‐bonding networks in the second coordination sphere. The tetra‐amide pendant arms of Eu^II^ **1** create an organized and sterically confined second‐coordination sphere that other lanthanide complexes of tetra‐phosphonates or nonmacrocyclic amides lack. The slow water exchange of the cyclen tetra‐amide moiety in conjunction with the hydrogen‐bonding capacity of the phosphonates in Eu^II^ **1** is responsible for the resistance to oxidation by O_2_. In addition to summarizing other reports that support the claim, we leveraged the ligand to report the mechanism of Eu^II^ **1** oxidation in response to oxygen exposure. Noninteger rate orders were observed when cuvettes containing Eu^II^ **1** were sparged with concentrations of gas that ranged from representative of hypoxic environments to massive excesses of oxygen, indicating complex reaction conditions that include reactive byproducts and multiple species of Eu^II^ **1**. Computational studies indicated that Eu^II^ **1** resistance to oxidation is due to the formation of hydrogen bonds between deprotonated phosphonate arms and a second‐sphere water molecule that blocks the exchange of inner‐sphere water that occupies the lone binding site for water of Eu^II^ **1**. Conversely, partially deprotonated forms of the ligand contain phosphonate arms hydrogen‐bound in conformations that enable the exchange of water and, consequently, the approach of oxygen to the inner‐sphere of Eu^II^. These results suggest that the unique persistence of Eu^II^ **1** in the presence of oxygen compared to other Eu^II^‐containing complexes is due to kinetic considerations of oxygen approach caused by hydrogen bonds between the phosphonate arms and second‐sphere water molecules. The results presented here lead to an understanding of the mechanisms of oxidation of Eu^II^ **1**, serve as a basis for quantifying hypoxia using Eu^II^ **1**, and provide useful information for the design of future Eu^II^‐based systems for the imaging of hypoxia or other redox‐active platforms within discrete complexes. Additionally, we presented computational modeling that is a robust tool for modeling the second‐sphere coordination environments of other systems involving the privileged cyclen tetra‐amide ligand.

## Conflicts of Interest

The authors declare no conflicts of interest.

## Supporting information



The authors have cited additional references within the Supporting Information [60, 61, 62, 63, 64, 65, 66, 67, 68, 69, 70, 71, 72, 73].
