# Supplementary material for: Cyclen Tetra‐Amide Ligands as a Privileged Class of Ligands for Studying Second‐Sphere Coordination Environments
Source: Chemistry. 2026 May 15;32(32):e71136. doi: 10.1002/chem.71136 (PMC13505747; doi:10.1002/chem.71136)
Supplement: Supplementary file 1 [file CHEM-32-e71136-s001.pdf]

Supporting Information

Cyclen tetra-amide ligands as a privileged class of ligands for studying second-sphere coordination environments.

Andrea L. Batchev,[a] Widana Kaushalya,[a] Chashitha Padukka,[a] Janya Subasinghe,[a] Alice R. Walker, [a,*] and Matthew J. Allen [a,*]

a. A. L. Batchev, W. Kaushalya, C. Padukka, J. Subasinghe, A. R. Walker, and M. J. Allen
Department of Chemistry, Wayne State University, Detroit, Michigan 48202, United States.
*E-mail: arwalker@wayne.edu and mallen@wayne.edu

Table of Contents

Experimental Procedures ***S2***

Representative UV–Visible Spectra of Time Courses ***S5***

Steady-State Approximations ***S12***

Gas Apparatus ***S13***

Computational model water distances to the Eu^{II} ion. ***S14***

Computational modeling of O_2 with inner- and second-sphere water molecules. ***S16***

Experimental Procedures

General Methods. Unless otherwise specified, all procedures were performed with the exclusion of oxygen using standard Schlenk and glovebox techniques, and all samples were prepared in a wet glove box (water is present but molecular oxygen is absent).[60] All solvents used were degassed under reduced pressure.

Commercially available chemicals were of reagent-grade purity and were used without further purification. Eu^{II} tetraazazyclododecane 1,4,7,10-tetrakis-acetamido-methylenephosphonic acid (Eu^{II}**1**) was prepared following a reported procedure.[40] Water was obtained from an ELGA PURELAB Ultra Mk2 water purifier. All UV–visible spectroscopy experiments, except for those involving the addition of superoxide, were performed using a stock solution of Eu^{II}**1** in 3-(N-morpholino)propanesulfonic acid buffer. The buffer was prepared by diluting commercially available 3-(N-morpholino)propanesulfonic acid [Fischer Scientific, 10X] to one-tenth of if its initial concentration, adjusting the pH to 7.5 using aqueous NaOH (1 M), and bringing the final volume to 2.000 mL with water. All values of pH were measured using pH indicator strips (Mquant, pH range 0–14, 1 pH unit or Mquant, pH range 2.0–9.0, 0.5 pH unit). O_2 calibration gases (0.01, 0.05, 2, 5, 8, 10, 12, 15, 18, 20.9, 23.4, and 30%) in nitrogen were obtained from CalGas Co. Gas containing 0% O_2 was obtained from house N_2. 2',7'-Dichlorofluorescein was obtained from Acros Organics, and potassium superoxide was obtained from SigmaAldrich. Concentrations of Eu were determined using energy-dispersive X-ray fluorescence spectroscopy at the Lumigen Instrument Center in the Department of Chemistry at Wayne State University. Calibration curves were created using the Eu isotope ion count for a concentration range of 0–1,000 ppm from diluted Sigma ICP standard solutions.

UV–Visible Time course Experiments for Oxygen Calibration Curves of Eu^{II}1. All UV–visible absorption spectra were acquired with a JASCO V-770 spectrophotometer. To study the persistence of Eu^{II}**1** after exposure to different amounts of oxygen, solutions of Eu^{II}**1** were prepared in a wet glove box by mixing 3-(N-morpholino)propanesulfonic acid buffer (10×, 200 mL), water, and an aqueous stock solution of Eu^{II}**1** to make final samples that contained Eu^{II}**1** in buffer (3.5 mM, 2.000 mL, pH = 7.5). Samples were placed in quartz cuvettes and capped with screw tops containing septa. The capped cuvettes were sealed with paraffin wax to prevent contamination from the outside atmosphere. An apparatus consisting of a three-necked flask was constructed to contain gas with desired concentrations of oxygen (0, 0.01, 0.05, 2, 5, 8, 10, 12, 15, 18, 20.9, 23.4, and 30% O_2 in N_2) (Supporting Information Figure S14). One stopcock was attached to vacuum, and the other stopcock was connected to a gas cylinder containing the desired concentration of oxygen. The third neck was fitted with a new, unpierced rubber septum. The flask was evacuated under vacuum and the rubber septum was pierced with a needle (22 Gauge) attached to a syringe (50 mL). The evacuated flask was filled with the desired gas, and the filling and evacuating steps were repeated a total of three times before a final filling with the desired gas for the experiment. The stopcocks were closed, and the flask was removed from the Schlenk line. UV–visible time course scans of Eu^{II}**1** were measured with the parameters of UV–visible bandwidth of 1.0 nm and near-IR bandwidth of 4.0 nm with absorbance measured at λ = 420 nm for 3,600 s. A blank was measured containing a solution of water and 3-(N-morpholino)propanesulfonic acid buffer before each scan. The sealed cuvettes of Eu^{II}**1** were placed in the UV–visible sample holder, and the initial absorbance each sample was recorded. Before sparging the cuvette containing Eu^{II}**1**, the syringe was filled with the desired gas. As the syringe was removed from the flask, slight pressure was exerted on the plunger until the syringe pierced the cuvette septum and entered the sample. Pressure was then increased to the rate of 1 mL s^{-1}. After sparging, the syringe and purging needle were immediately removed, and electrical tape was placed over the top of the septum to prevent atmospheric air from entering the cuvette through the hole made by the syringe. The time course was started, and the time it took between piercing the septum and starting the time course was recorded.

7,10-Dichlorofluoresceine Time course UV–Visible Measurements. In the glovebox, 7,10-dichlorofluoresceine (2 mg, 4 mmol) was dissolved in degassed water (5 mL). The resulting solution (50 mL) was used to prepare samples of Eu^{II}**1** (3.5 mM) and 3-(N-morpholino)propanesulfonic acid buffer (10X, 200 mL). Degassed water was added to bring the final volume to 2 mL. The quartz cuvettes were closed with a screw-top cap containing a septum and sealed with parrafin wax. A single UV–visible absorption scan from 300 to 700 nm was performed with the cap of the cuvette closed (t = 0). The sample was sparged with either air or O_2 (8% in nitrogen) as described in the UV–visible time course section. The time course absorbance measurements were acquired at λ = 500 nm for 3,600 s. Parameters for the scan included a scan rate of 1,000 nm min^{-1}, UV–visible bandwidth of 1.0 nm, and near-IR bandwidth of 4.0 nm.

Superoxide Experiments. Samples of Eu^{II}**1** (3.68 mM, 1.900 mL) containing 3-(N-morpholino)propanesulfonic acid buffer (10X, 200 mL) were prepared in paraffin-wax-sealed quartz cuvettes as described in the UV–visible time course section. A 1 mL syringe was filled with a solution of potassium dioxide (0.1 mL, 0.5 mg, 7 mmol) in water, closed, and sealed with parrafin wax. The initial absorbance of each Eu^{II}**1** solution was recorded at λ = 420 nm. A stopwatch was started, then the potassium dioxide was quickly injected into the cuvette containing Eu^{II}**1**. The cuvette was shaken lightly, and the time course measurements were started. The time taken to inject the potassium dioxide into the cuvette and start the measurement was recorded. The parameters for the scan were the same parameters used in the oxygen calibration curve.

Headspace Experiments. Samples containing Eu^{II}**1** in buffer (3.5 mM, 2.000 mL, pH = 7.5) were prepared and placed in quartz cuvettes containing septum caps and sealed with paraffin wax as described in the UV–visible time course section. The initial absorbance of each sample of Eu^{II}**1** was recorded before sparging. The septum cap to the cuvette was simultaneously pierced with a purge needle and a syringe used to sparge 50 mL of air into the headspace of the cuvette at a rate of 1 mL s^{-1}, without breaking the surface of the liquid. After sparging, both needles were removed from the septum simultaneously, electrical tape was quickly placed over the septum, and time course measurements were started (λ = 420 nm). The UV–visible spectroscopy parameters were the same as for those used in the oxygen calibration curve time course experiments.

Geometry Optimizations. Quantum-mechanical geometry optimizations of gas-phase and solution-states using implicit water molecules were performed with the Gaussian 16 program.[61] The structures of [Eu^{II}**1**]$^{6-}$ and [Eu^{II}**1**H_4]$^{2-}$ were calculated by modifying a similar Eu^{II} complex from the Cambridge Crystallographic Data center (Deposition number: CCDC 1562330).[37] [Eu^{II}**1**H]$^{5-}$ geometry optimization was achieved by adding a proton to the [Eu^{II}**1**]$^{6-}$ optimized structure. The structures were optimized using density functional theory with the hybrid B3PW91 functional, with the Eu^{II} ion treated with the Stuttgardte–Dresden relativistic core potential as reported elsewhere and the remaining atoms were treated with a 6-31g basis set.[62,63] The 54 atoms in common between Eu^{II}**1** and the reported structure were frozen in the positions of the crystal structure to minimize computational time.

Classical Molecular Dynamic Simulations. We performed classical molecular dynamics simulations on europium-ligand complexes in three protonation states: [Eu^{II}**1**]$^{6-}$, [Eu^{II}**1**H]$^{5-}$, and [Eu^{II}**1**H_4]$^{2-}$. Each system was solvated in a TIP3P water box,[64] ensuring a distance of 18 Å between the solute and the boundary of the solvent box. Sodium cations were added to neutralize the net charge of each system to zero (six sodium cations for the fully deprotonated form, five for the monoprotonated form, and two for the half-protonated form). Topology and coordinate files were generated using the tleap module from AMBER20.[65,66] Custom parameters for each complex were generated with AutoParams,[67] which fit restrained electrostatic potential charges[68] from the optimized gas-phase geometries as described in the previous section, and provided geometric parameters based primarily on OL15 force field parameters.[69] The divalent Eu^{II} Li and Merz 12-6-4 TIP3P parameters in Amber were used to model the europium ion.[70,71] Additionally, modifications to the default oxygen angle phosphate parameters were incorporated to maintain the stability of the

terminal phosphates during the simulations.[72] After energy minimization and equilibration, production molecular dynamics simulations were performed for 100 ns and 1,000 ns with short replicate simulations for experiments without and with O_2, respectively, in the constant-pressure, constant-temperature ensemble using a timestep of 1 fs. Periodic boundary conditions were applied with the temperature maintained at 300 K using a Langevin thermostat (collision frequency δ = 5 ps^{-1}) and pressure maintained at 1 atm. A 12 Å cutoff was used for nonbonded interactions. During the simulation of the monoprotonated Eu complex, a distance restraint was applied to the Eu atom to maintain its initial position. During the simulation of the monoprotonated Eu complex, square harmonic restraining potential of 50 kcal/mol with a distance of 2.44–2.62 Å was applied to the Eu–N distance to maintain its initial position. The same constraints were applied to the fully deprotonated system in a replicate trajectory, but it did not alter the results. Calculations were also performed with an initial O_2 molecule bound to the Eu atom, where a square harmonic restraining potential of 10 kcal/mol·$Å^2$ was applied to the Eu–O distance to maintain the non-bonded interaction between the europium ion and molecular oxygen at an equilibrium distance of 2.510 Å. Force field parameters for molecular oxygen were adopted from a previous report.[73] Replicate simulations were performed without the Eu–O constraint, which did not alter the water exchange but did result in the rapid dissociation of O_2. All the other conditions were the same as the complexes without the oxygen molecule.

Representative UV–Visible Spectra of Time Courses (Supporting Figures S1–S13)

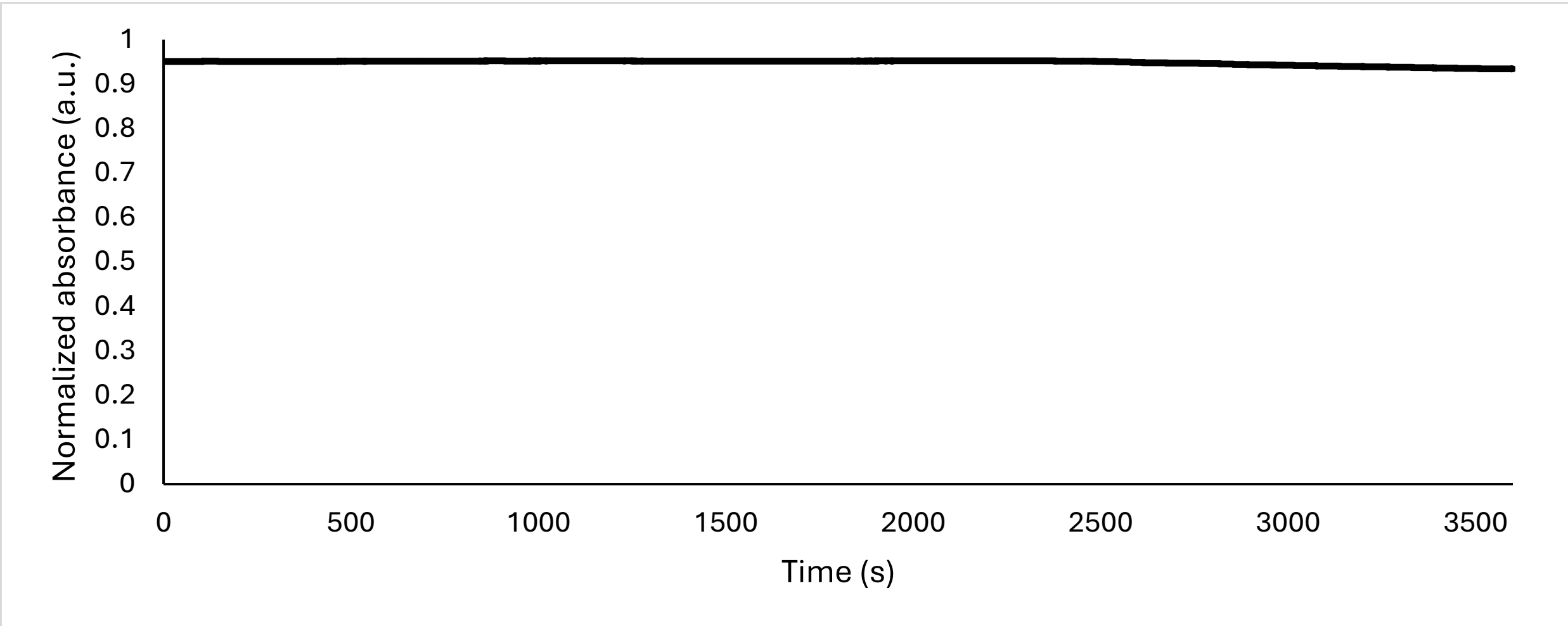

Supporting Figure S1. Representative UV–visible spectroscopy time course of Eu^{II}**1** (3.5 mM, 2.000 mL) sparged with 99.999% N_2 (50 mL).

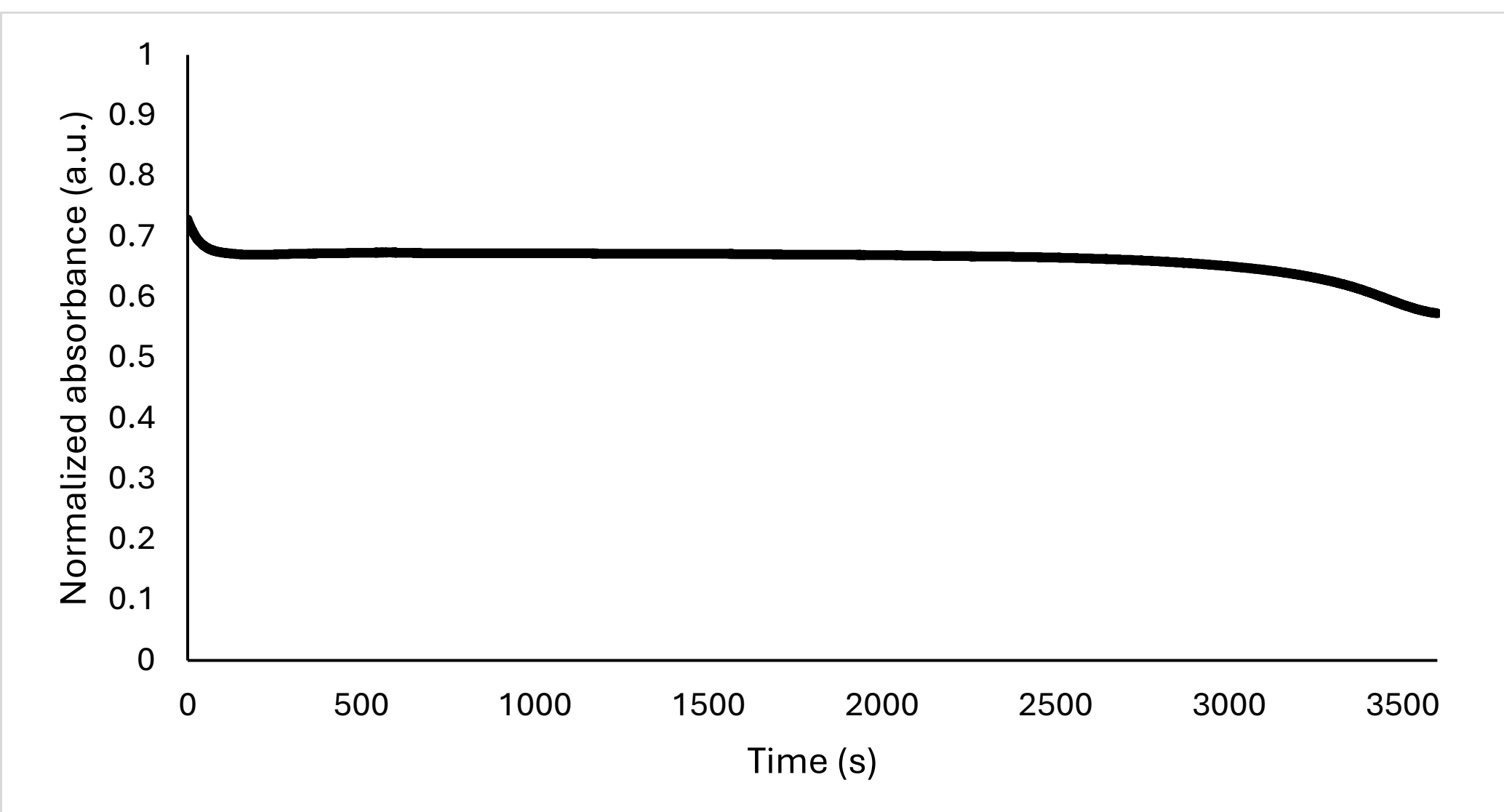

Supporting Figure S2. Representative UV–visible spectroscopy time course of Eu^{II}**1** (3.5 mM, 2.000 mL) sparged with 0.01% O_2 in N_2 (50 mL).

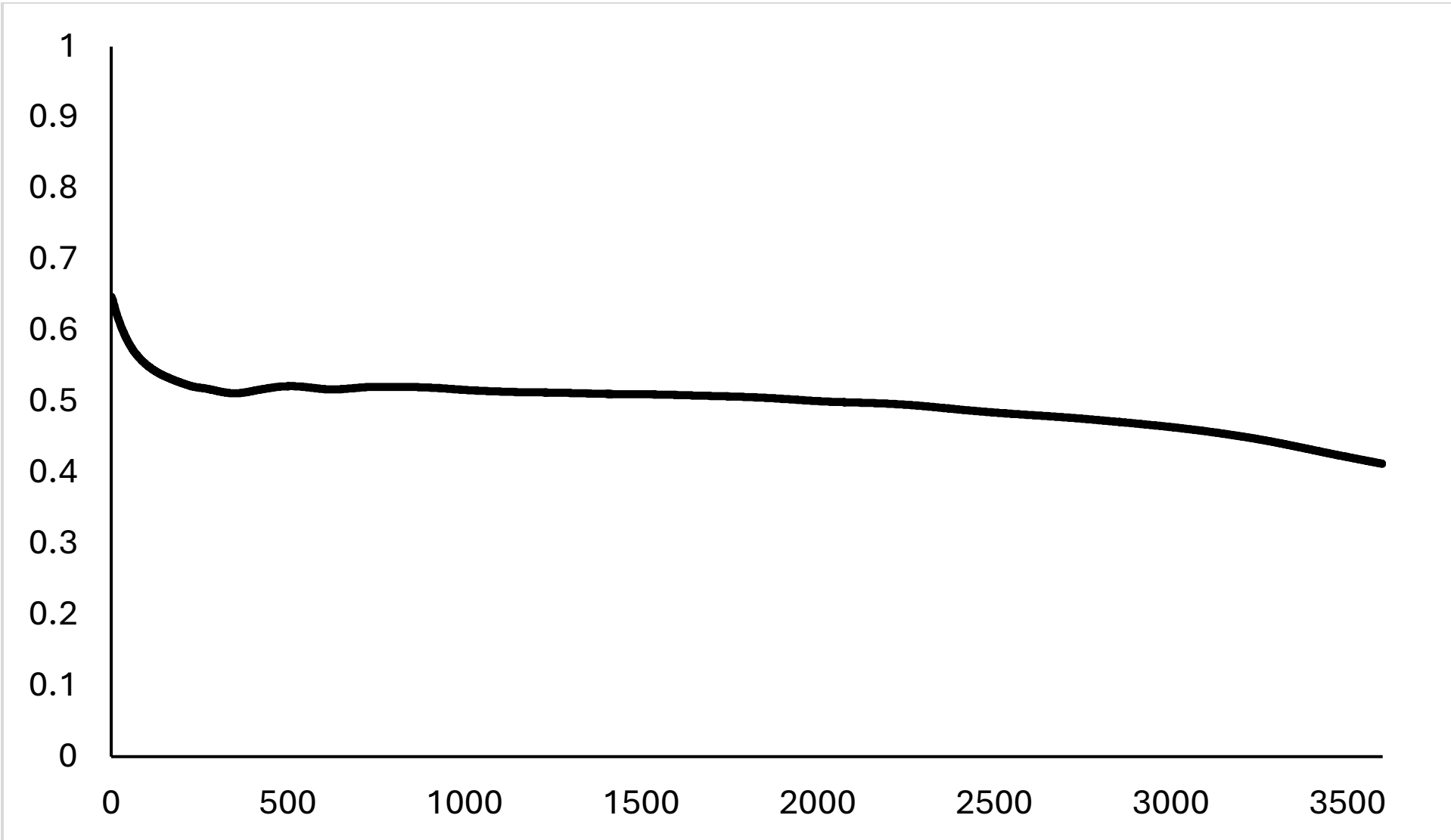

Supporting Figure S3. Representative UV–visible spectroscopy time course of Eu[II]**1** (3.5 mM, 2.000 mL) sparged with 0.05% O_2 in N_2 (50 mL).

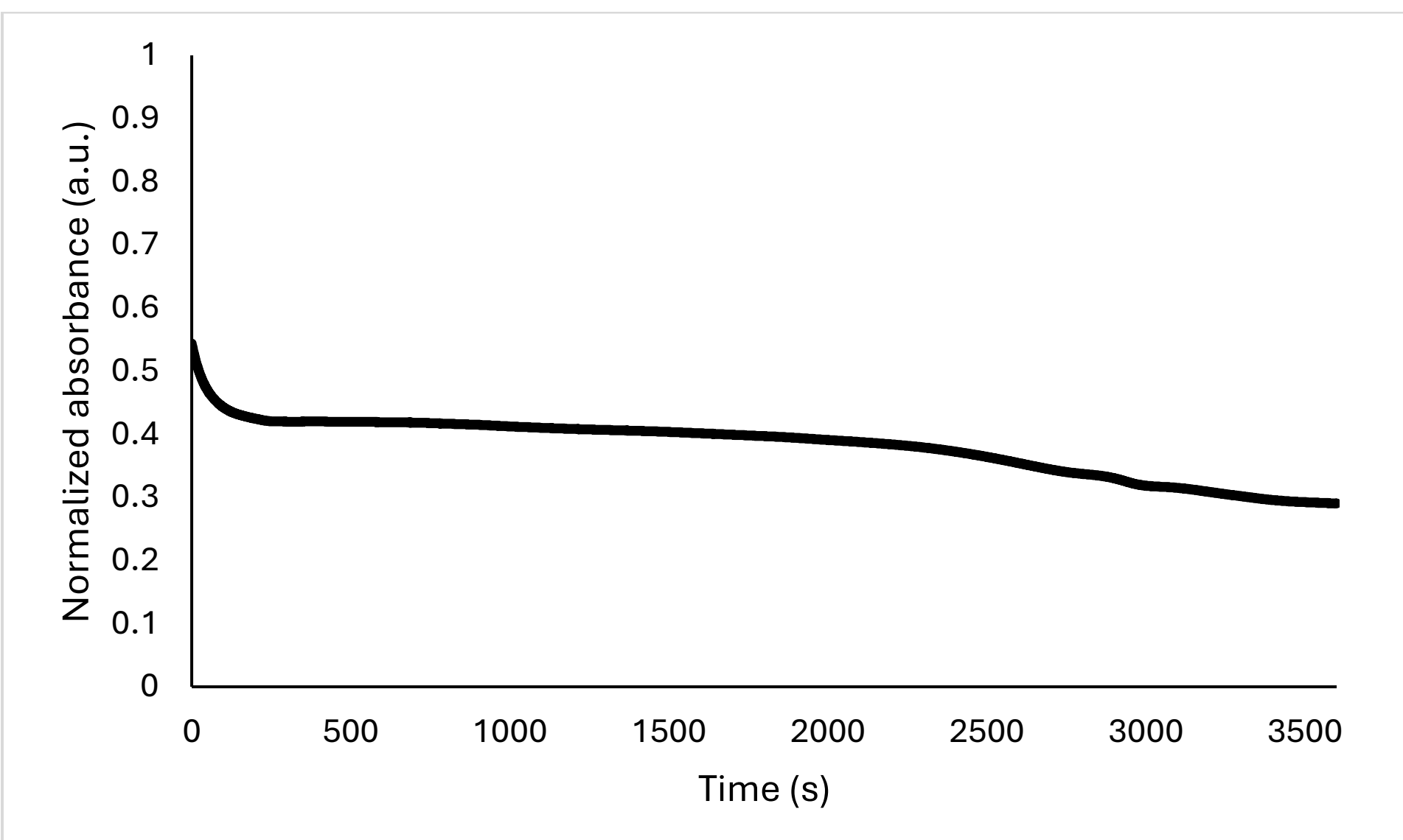

Supporting Figure S4. Representative UV–visible spectroscopy time course of Eu[II]**1** (3.5 mM, 2.000 mL) sparged with 2% O_2 in N_2 (50 mL).

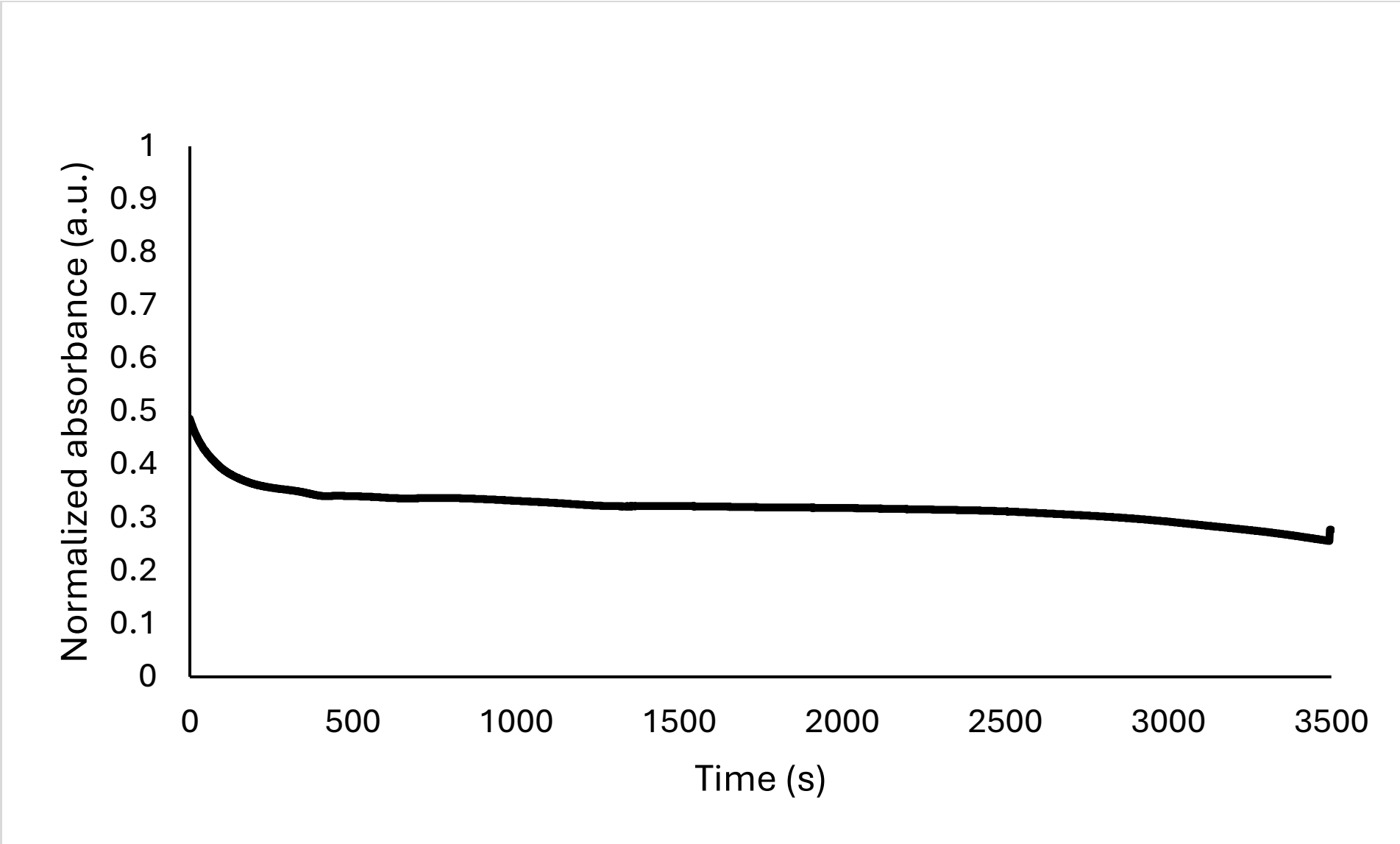

Supporting Figure S5. Representative UV–visible spectroscopy time course of Eu^{II}**1** (3.5 mM, 2.000 mL) sparged with 5% O_2 in N_2 (50 mL).

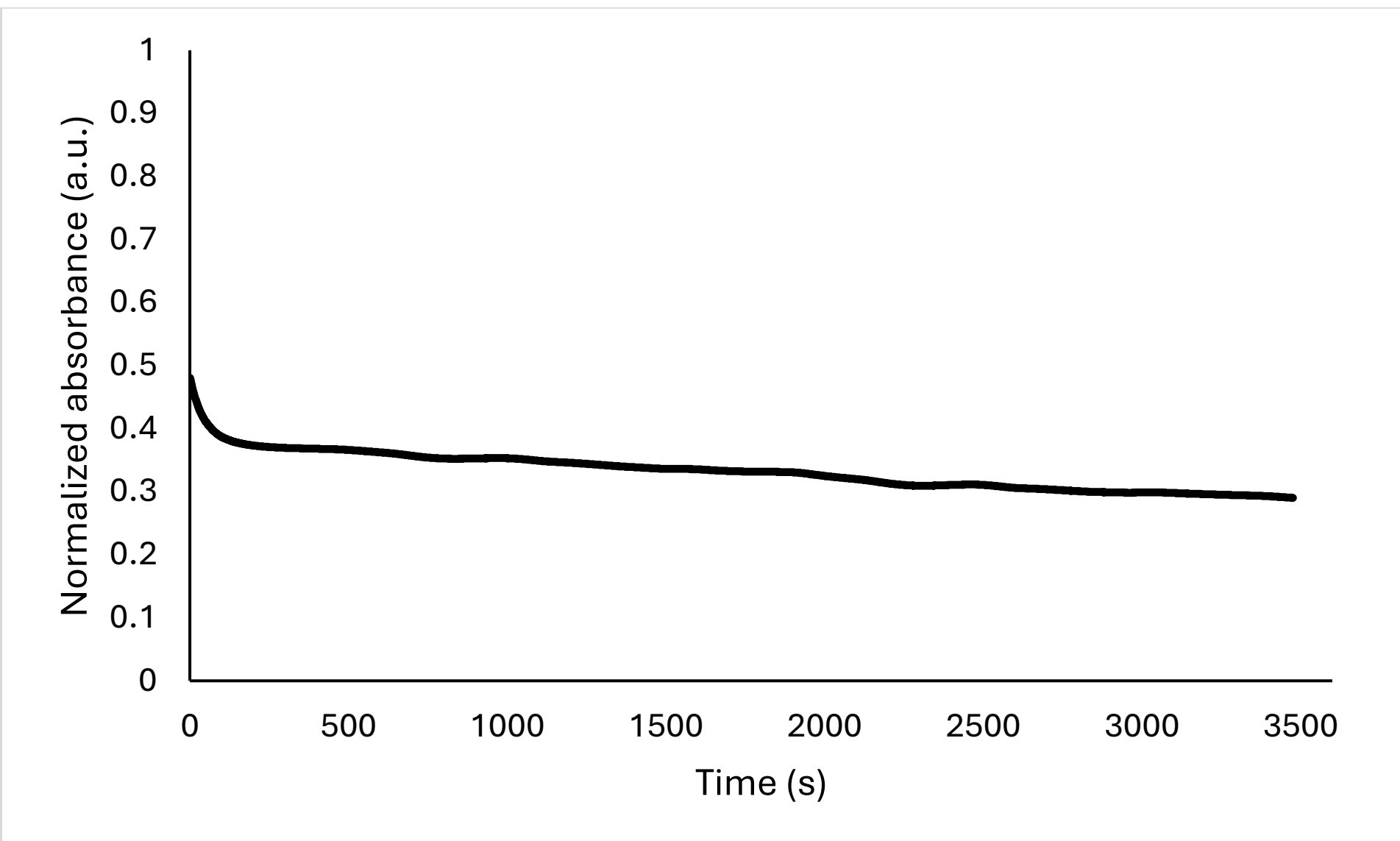

Supporting Figure S6. Representative UV–visible spectroscopy time course of Eu^{II}**1** (3.5 mM, 2.000 mL) sparged with 8% O_2 in N_2 (50 mL).

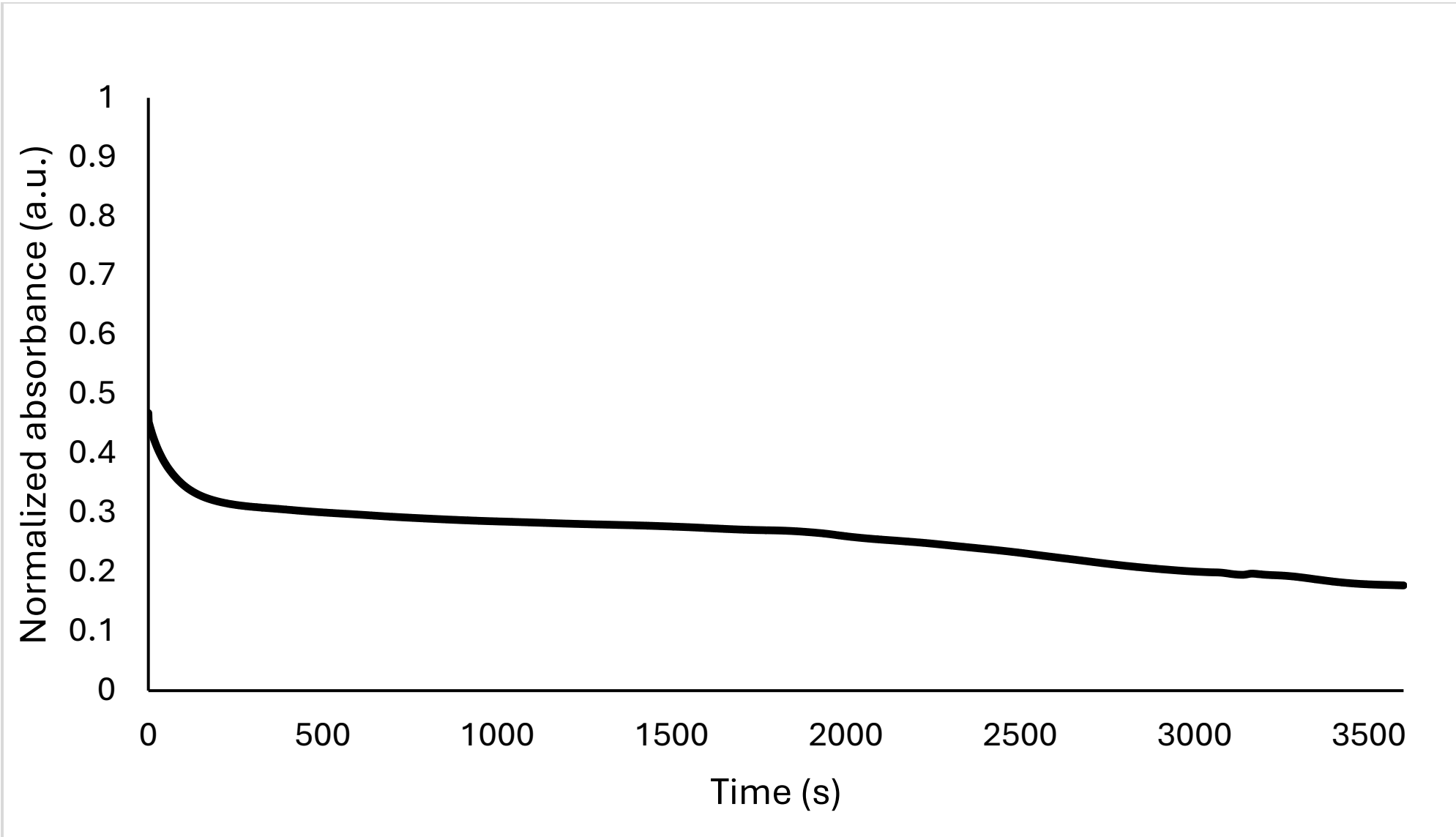

Supporting Figure S7. Representative UV–visible spectroscopy time course of Eu[II]**1** (3.5 mM, 2.000 mL) sparged with 10% O_2 in N_2 (50 mL).

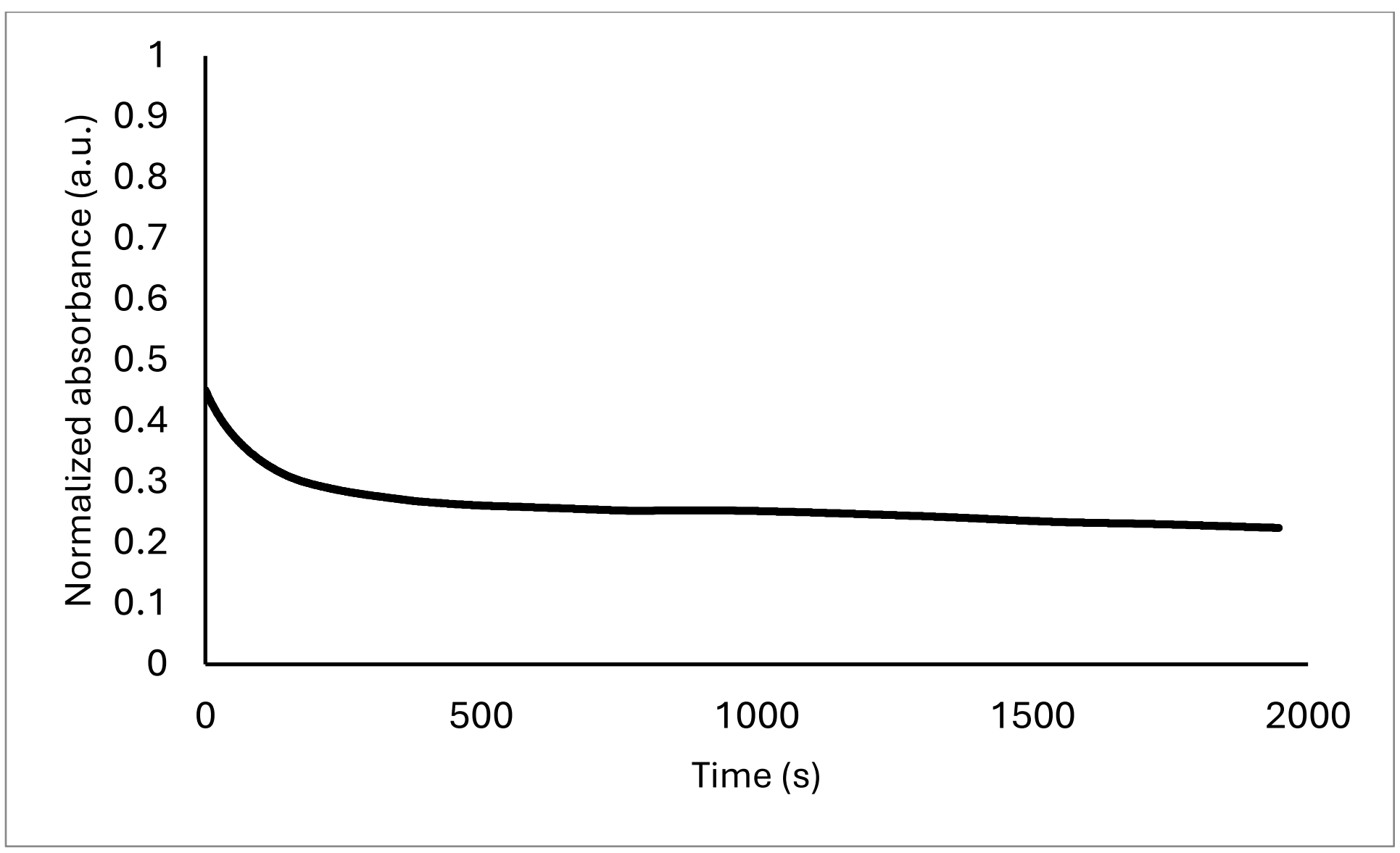

Supporting Figure S8. Representative UV–visible spectroscopy time course of Eu[II]**1** (3.5 mM, 2.000 mL) sparged with 12% O_2 in N_2 (50 mL).

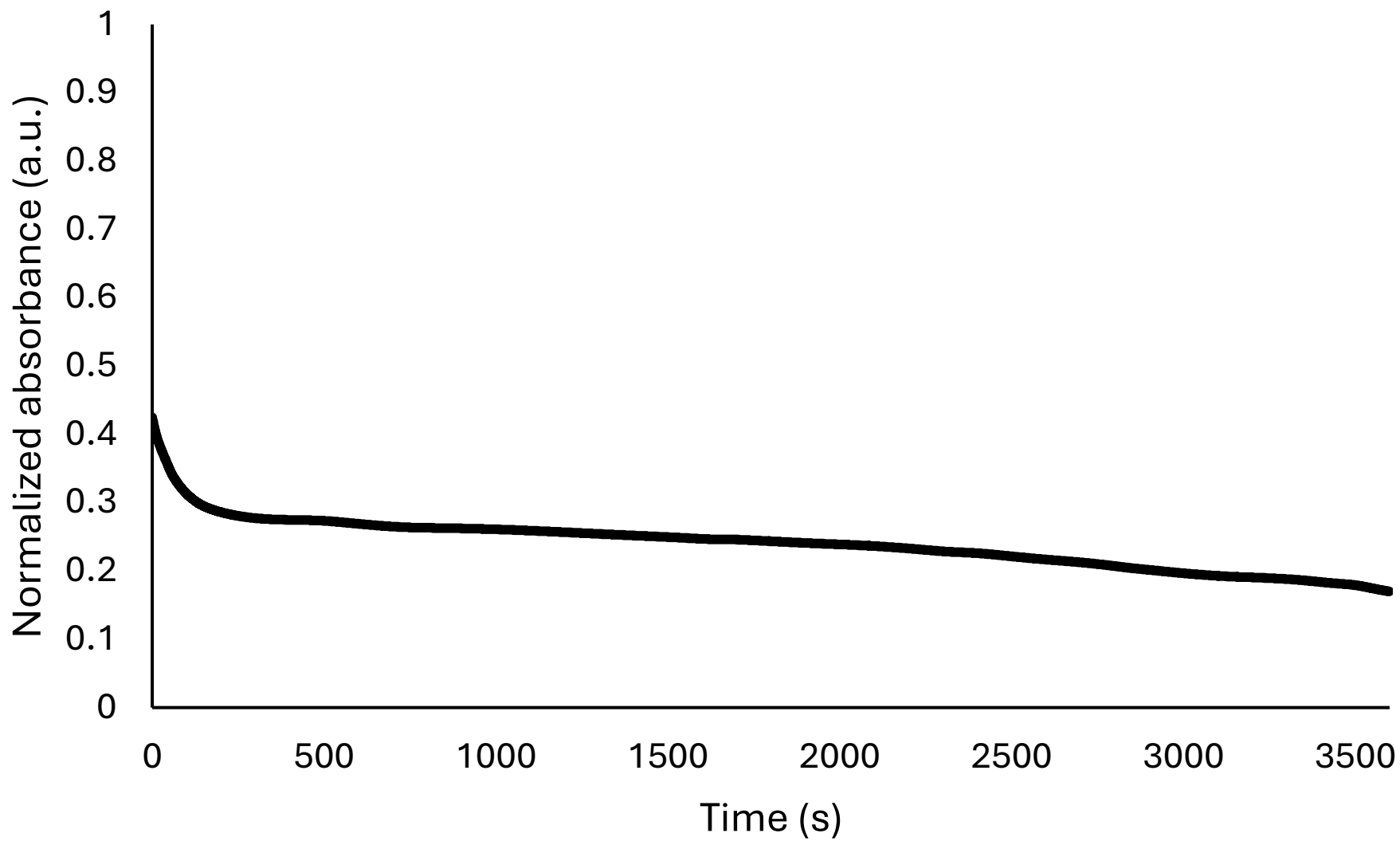

Supporting Figure S9. Representative normalized mean UV–visible spectroscopy time course of Eu^{II}**1** (3.5 mM, 2.000 mL) sparged with 15% O_2 in N_2 (50 mL).

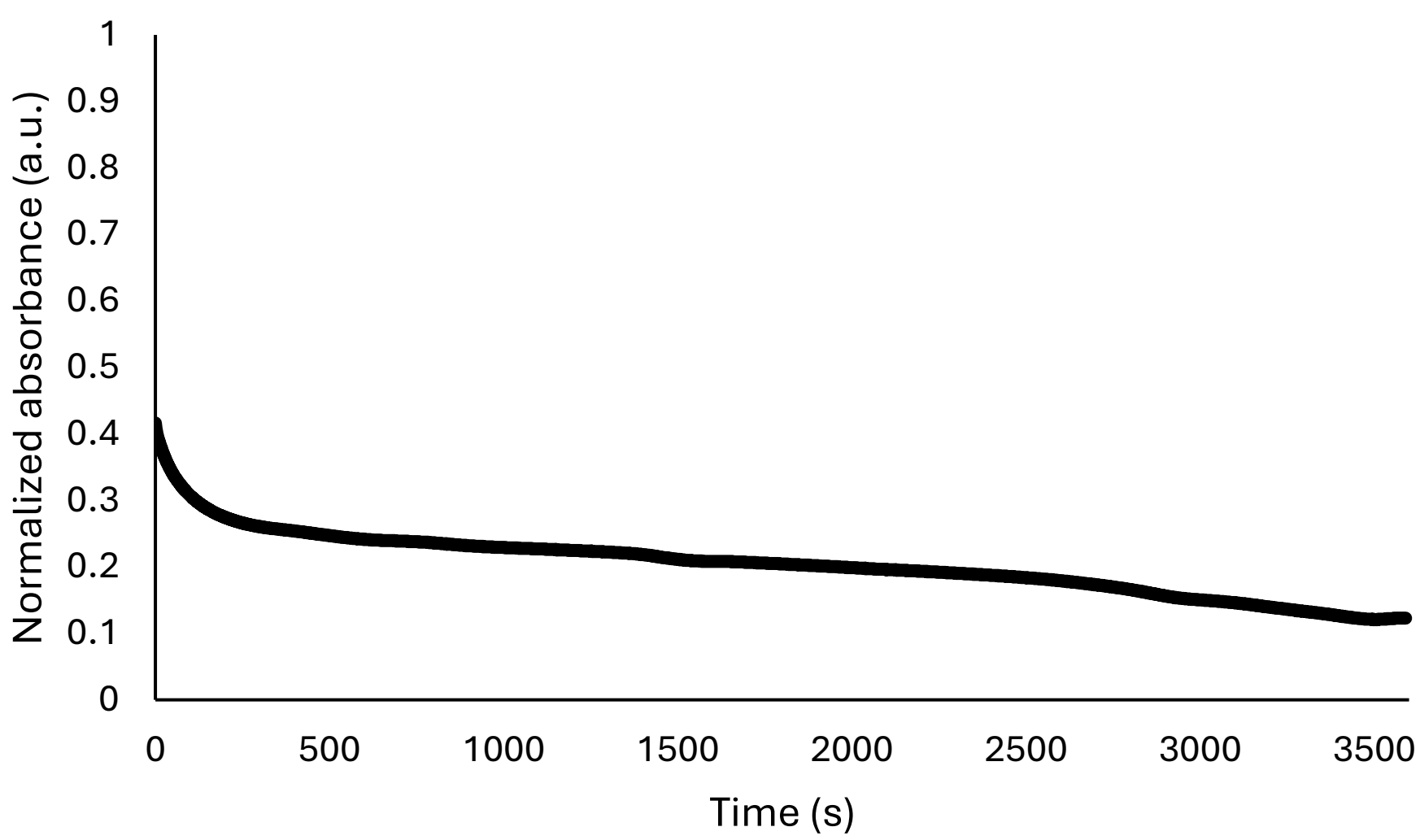

Supporting Figure S10. Representative normalized mean UV–visible spectroscopy time course of Eu^{II}**1** (3.5 mM, 2.000 mL) sparged with 18% O_2 in N_2 (50 mL).

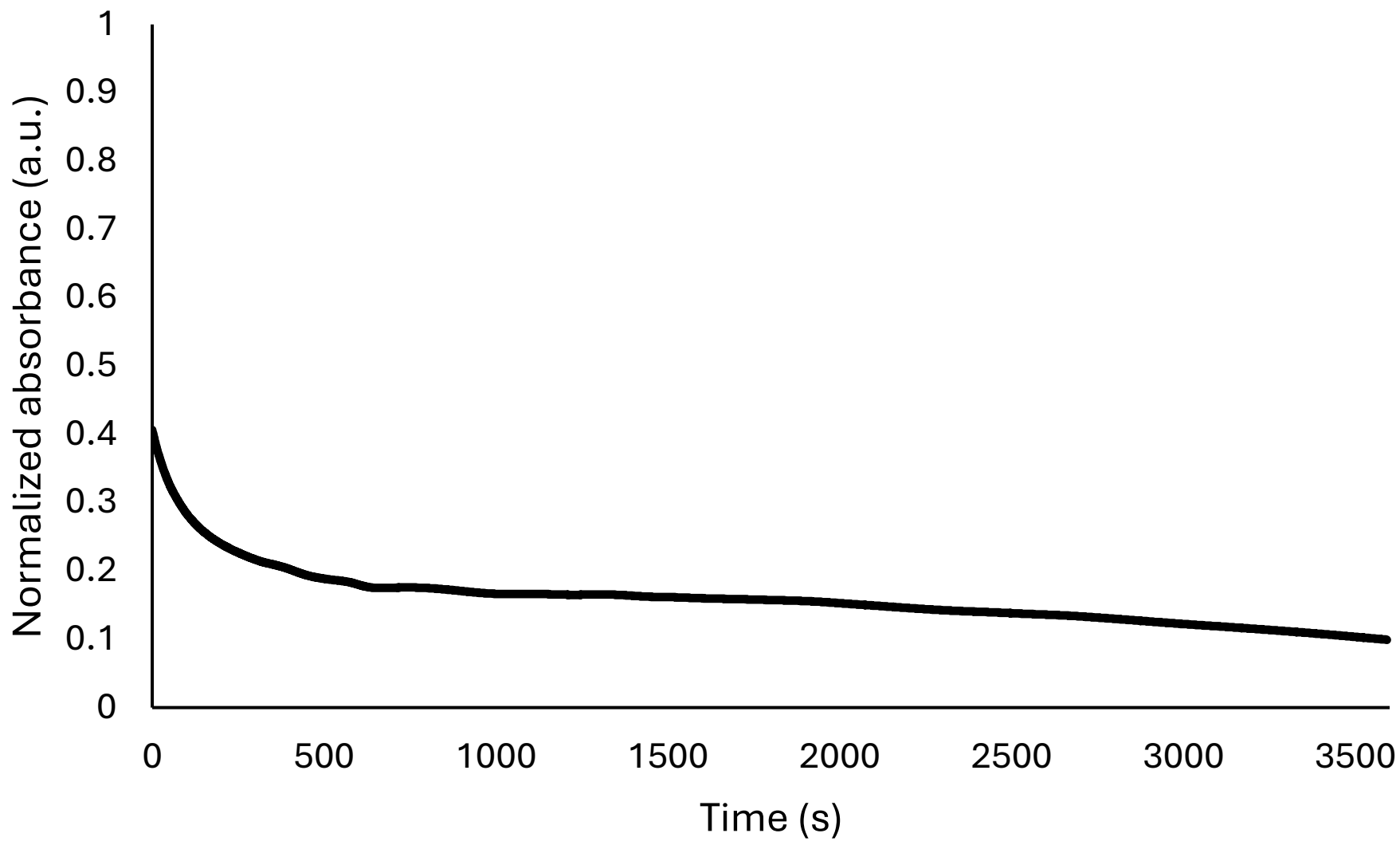

Supporting Figure S11. Representative normalized mean UV–visible spectroscopy time course of Eu^{II}**1** (3.5 mM, 2.000 mL) sparged with 20.9% O_2 in N_2 (50 mL).

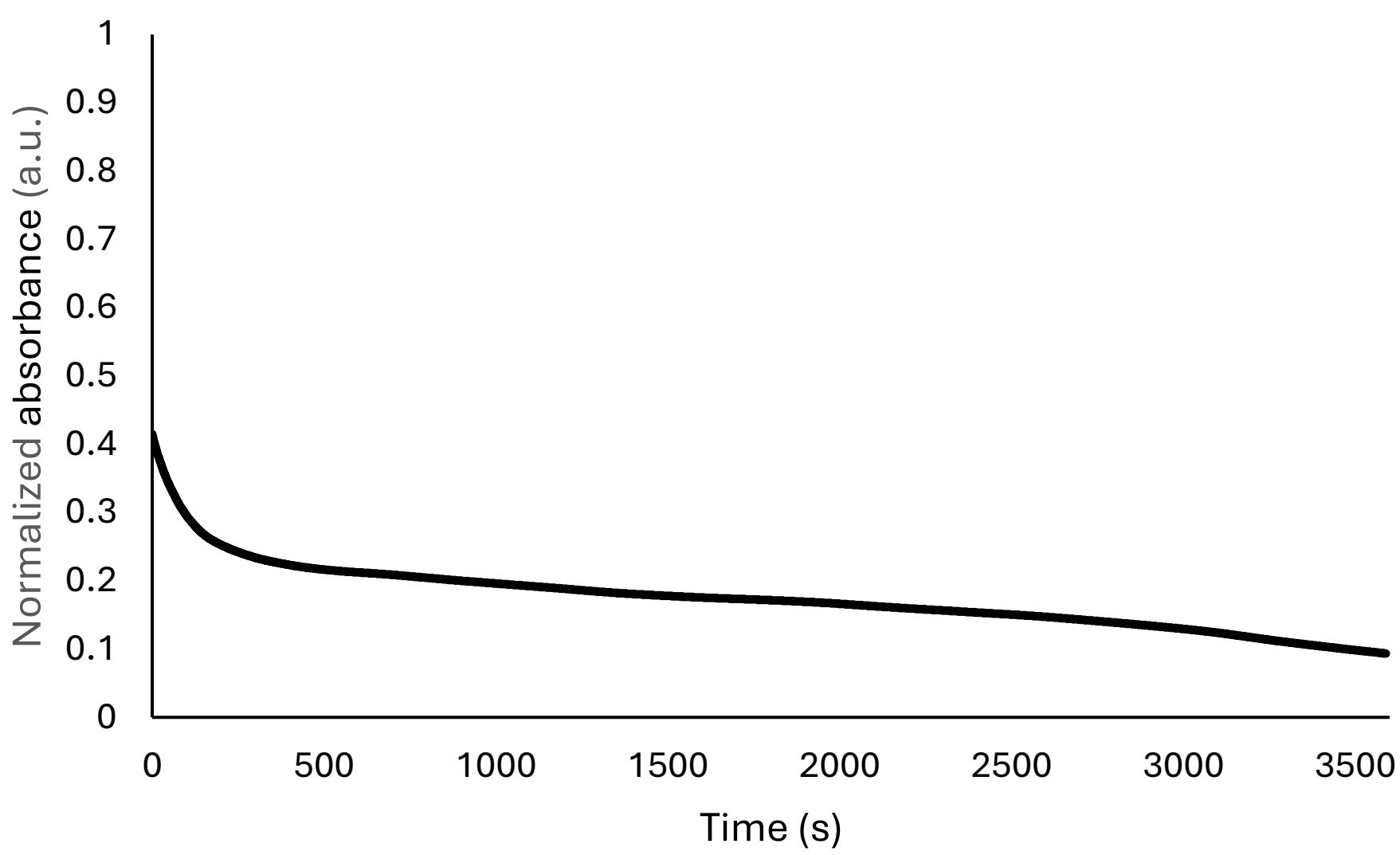

Supporting Figure S12. Representative normalized mean UV–visible spectroscopy time course of Eu^{II}**1** (3.5 mM, 2.000 mL) sparged with 23.4% O_2 in N_2 (50 mL).

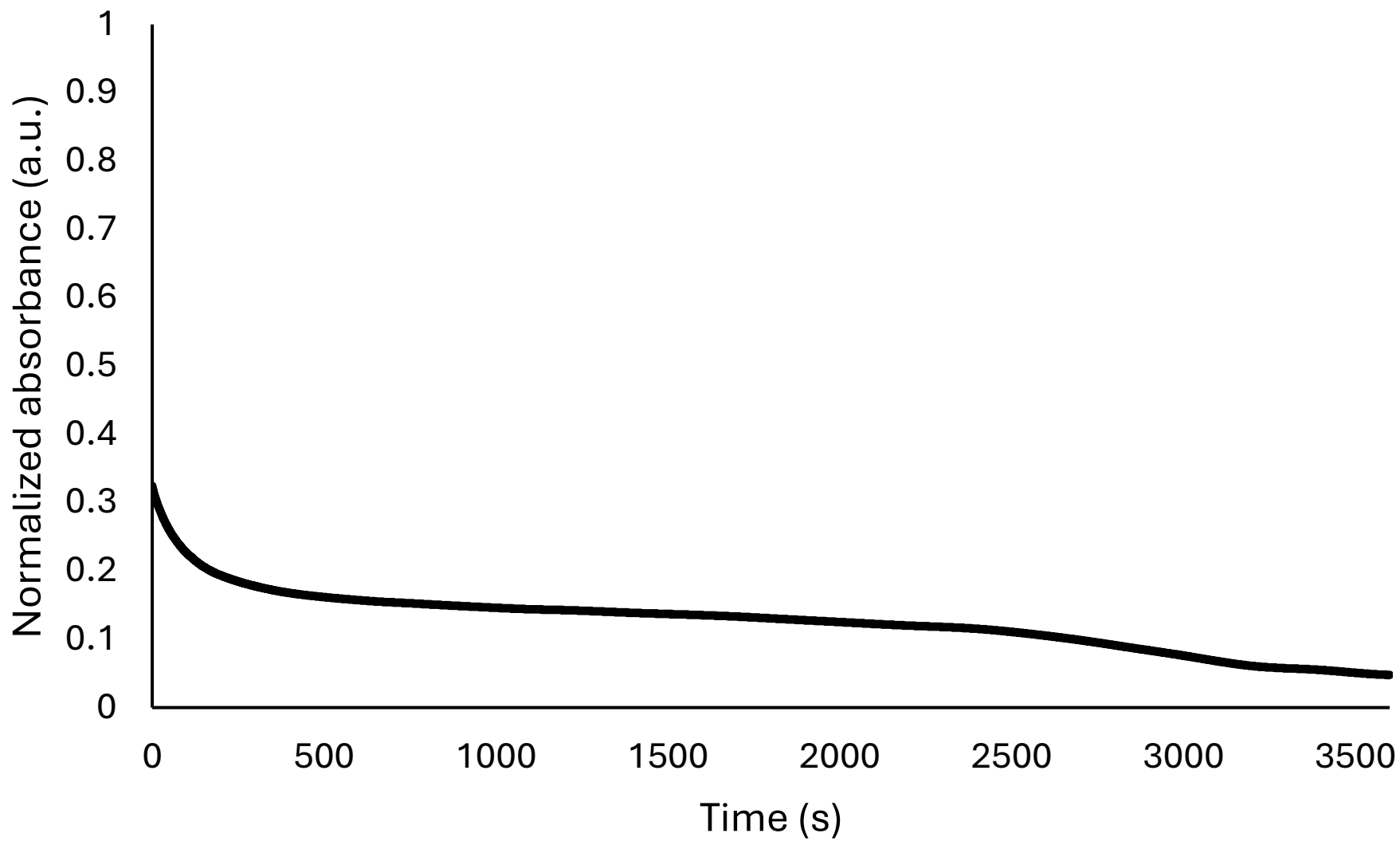

Supporting Figure S13. Representative normalized mean UV–visible spectroscopy time course of Eu^{II}**1** (3.5 mM, 2.000 mL) sparged with 30% O_2 in N_2 (50 mL).

Steady-State Approximations (Supporting Equations S1–S10)

The reaction rate of Eu^{II}**1** and O_2 can be derived from **Supporting Eqs. 1** and **2**.

Eq S1 $$Reaction\ Rate = \frac{d[Eu\mathbf{1}^{III}]}{dt} = -\frac{d[Eu\mathbf{1}^{II}]}{dt} = -\frac{d[O_2]}{dt}$$

Eq S2 $$Reaction\ Rate = k[O_2]^x[Eu^{II}\mathbf{1}]^y$$

Where k is the rate constant of the reaction and x and y are the reaction orders of O_2 and Eu^{II}**1**, respectively.

When the concentration of Eu^{II}**1** is at a great excess compared to O_2, then the concentration change in Eu^{II}**1** compared to that of O_2 can be practically estimated to 0 and can therefore be removed from the rate equation. The reaction order of O_2, x, at an excess of Eu^{II}**1** can then be approximated through **Supporting equations S3–S6**:

Eq S3 $$Reaction\ Rate = k[O_2]^x$$

k is experimentally found as the slope of the reaction rate, as measured by absorbance intensity, versus the concentration of O_2.

Eq S4 $$[O_2]^x = \frac{Reaction\ Rate}{k}$$

Eq S5 $$xln[O_2] = ln(\frac{Reaction\ Rate}{k})$$

Eq S6 $$x = \frac{[ln\left(\frac{Reaction\ Rate}{k}\right)]}{ln[O_2]}$$

To find the reaction order of Eu^{II}**1**, y, when the concentration of O_2 changes minimally compared to the concentration of Eu^{II}**1**, **Supporting Equations S7–S10** can be similarly derived:

Eq S7 $$Reaction\ Rate = [Eu^{II}\mathbf{1}]^y k$$

Eq S8 $$[Eu^{II}\mathbf{1}]^y = \frac{Reaction\ Rate}{k}$$

Eq S9 $$yln[Eu^{II}\mathbf{1}] = Ln\frac{Reaction\ Rate}{k}$$

Eq S10 $$y = \frac{[\ln\left(\frac{Reaction\ Rate}{k}\right)]}{n[Eu^{II}\mathbf{1}]}$$

Gas Apparatus (Supporting Figure S14).

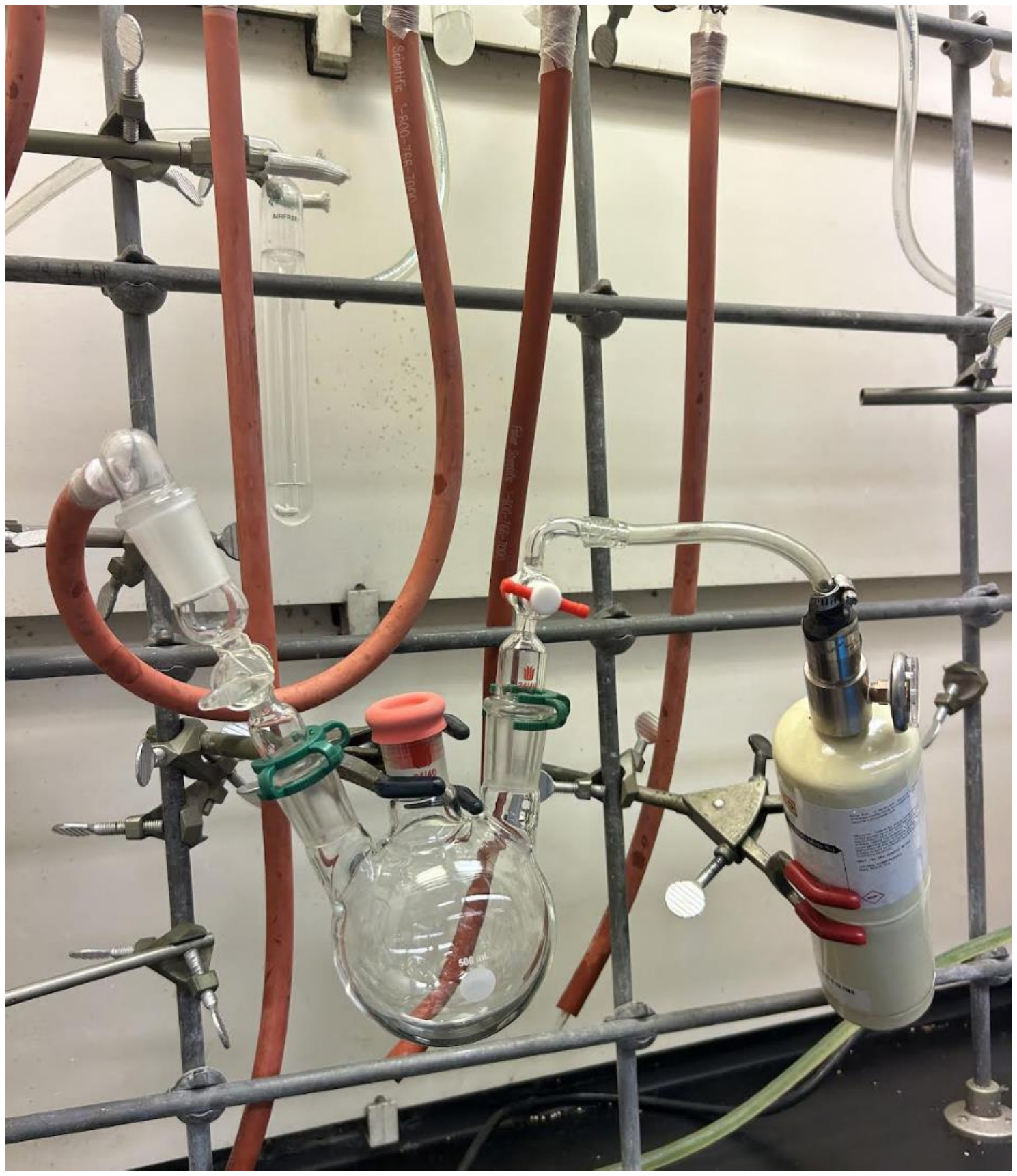

Supporting Figure S14. A 1 L three-necked flask was equipped with two different stopcocks. One stopcock was attached to vacuum, and the other stopcock was connected to a gas cylinder containing the desired concentration of oxygen. The third neck was fitted with a new, unpierced rubber septum.

Computational model water distances to the Eu^{II} ion (Supporting Figures S15–S18).

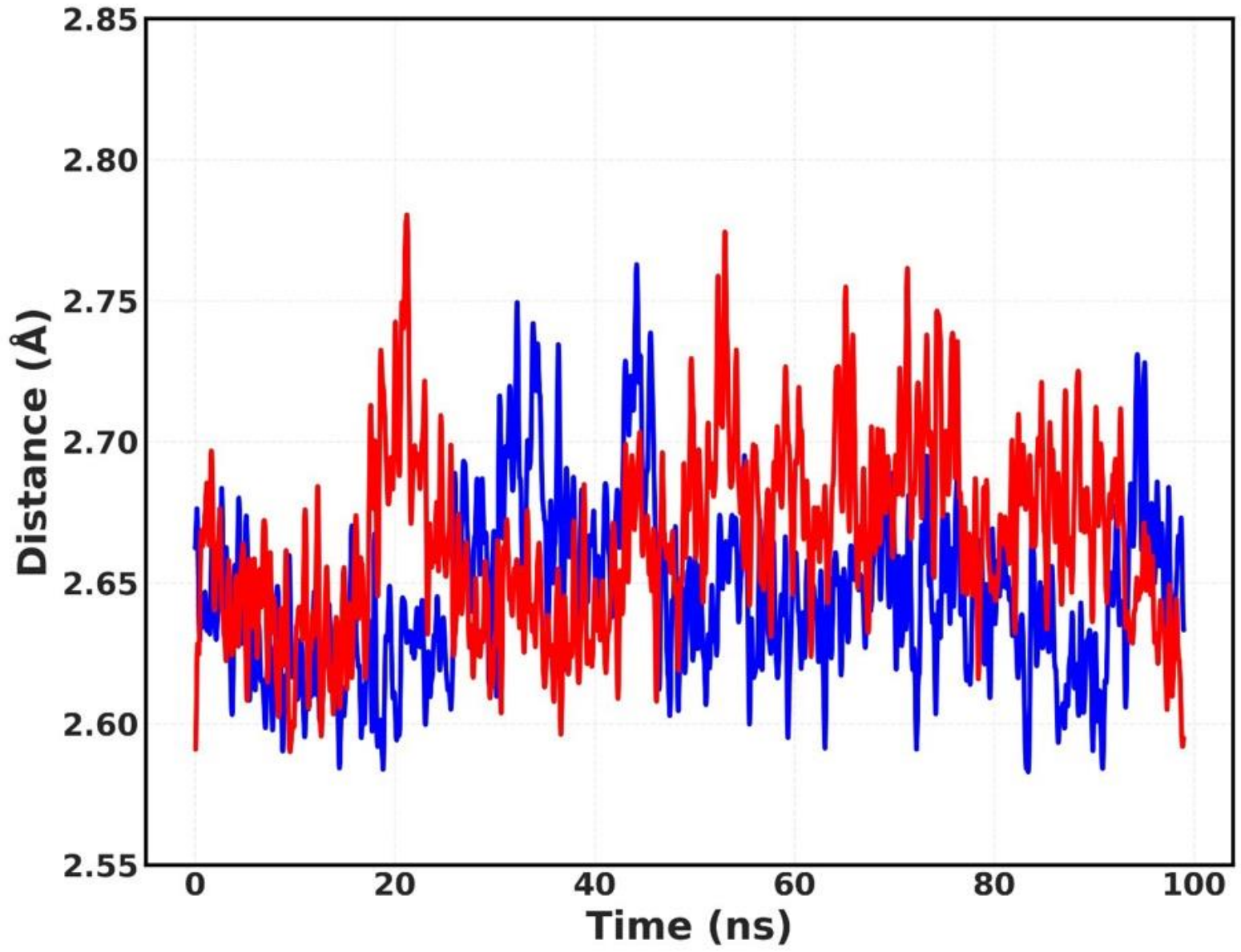

Supporting Figure S15. Distances to the Eu ion for inner- and second-sphere waters over time for the fully deprotonated Eu^{II}**1** species $[Eu^{II}\mathbf{1}]^{6-}$, where the red and blue lines represent the distances of two water molecules from Eu^{II} that exchange coordination between the inner- and second-sphere.

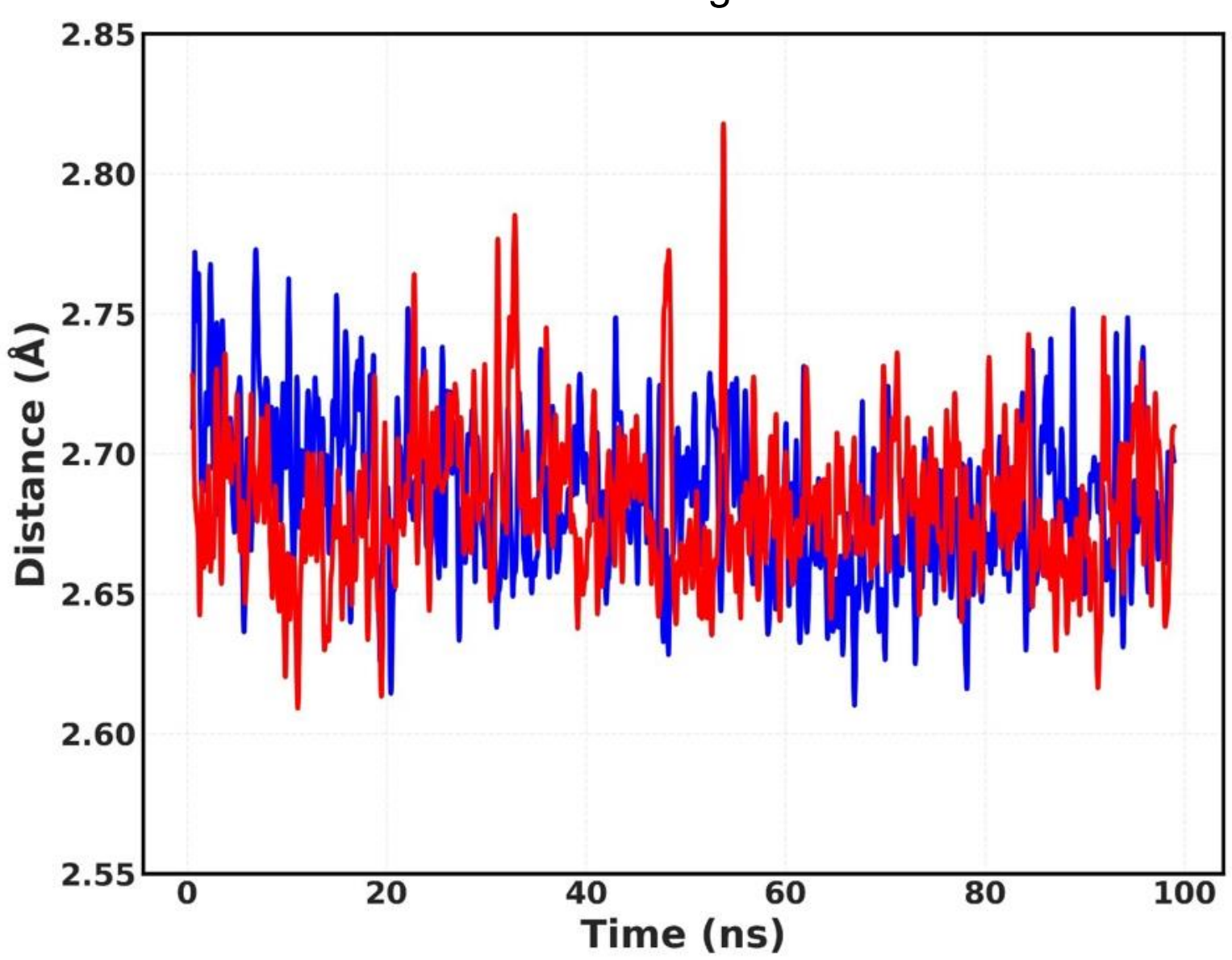

Supporting Figure S16. Distances to the Eu ion for inner- and second-sphere waters over time for the fully monoprotonated Eu^{II}**1** species $[Eu^{II}\mathbf{1}H]^{5-}$, where the red and blue lines represent the distances of two water molecules from Eu^{II} that exchange coordination between the inner- and second-sphere.

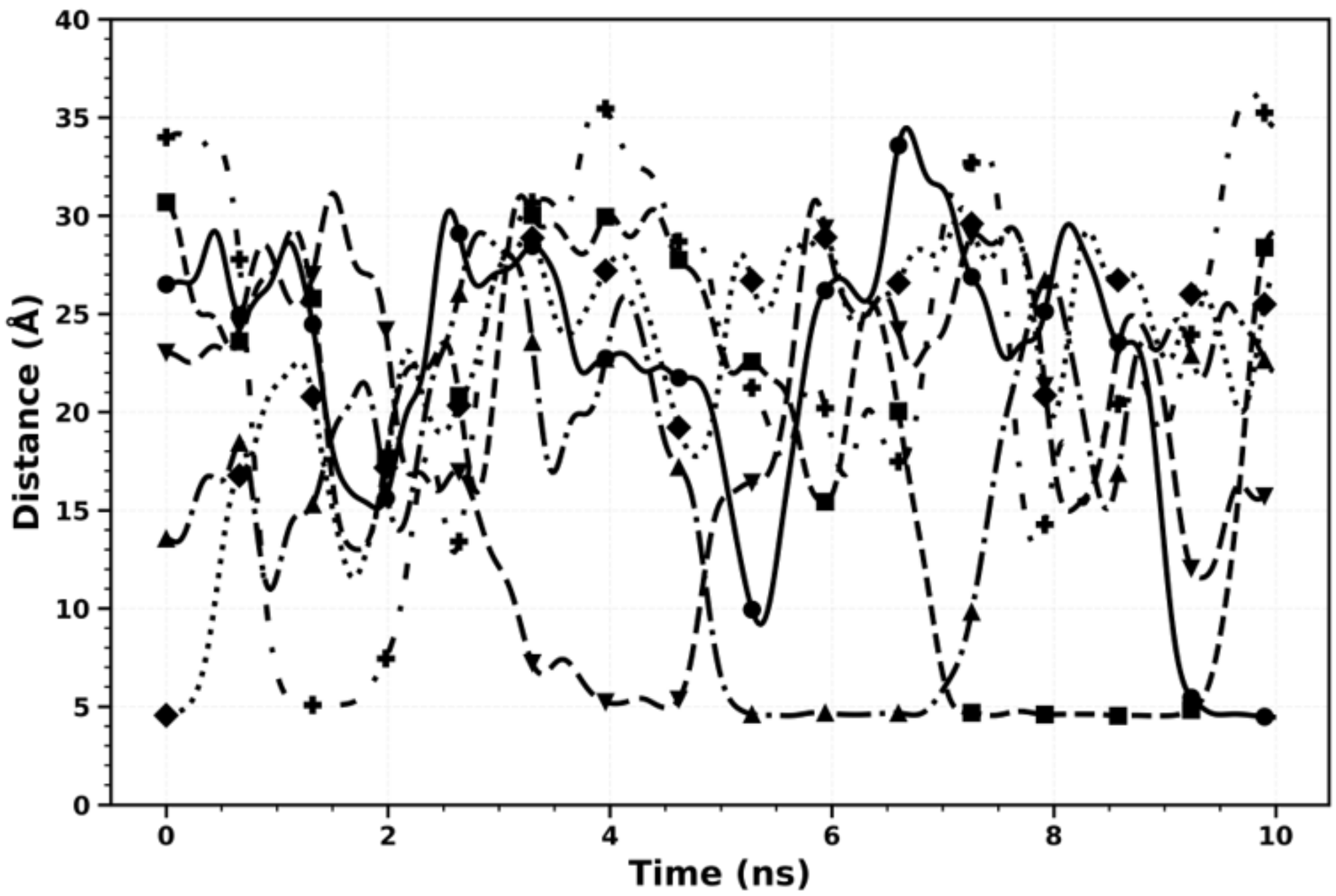

Supporting Figure S17. Distances to the Eu ion for second-sphere waters over time for the tetraprotonated Eu^{II}**1** species $[Eu^{II}\mathbf{1}H_4]^{2-}$. The different lines represent different water molecules that exchanged coordination spheres in the simulation.

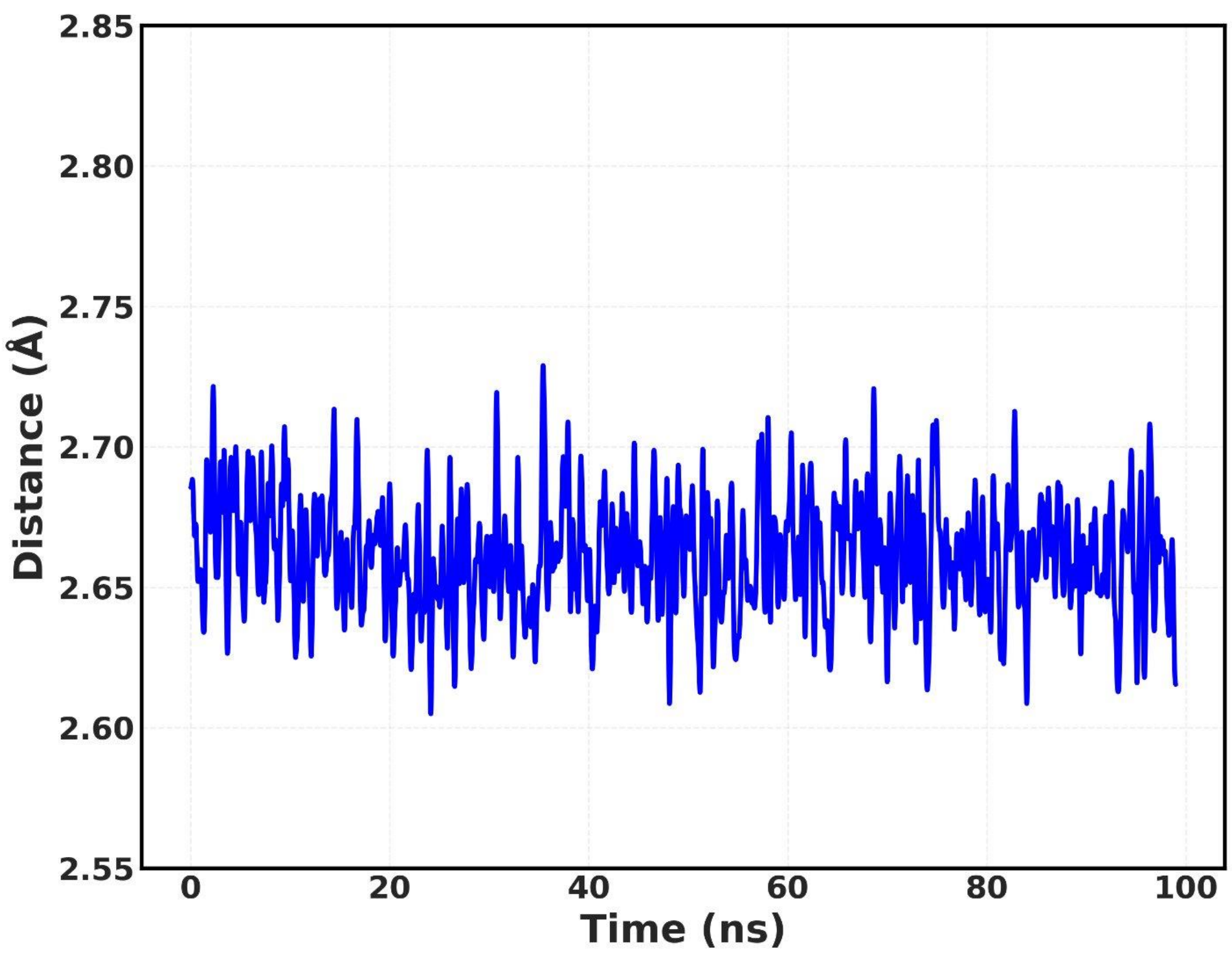

Supporting Figure S18. Distances to the Eu ion for inner-sphere water over time for the tetraprotonated Eu^{II}**1** species $[Eu^{II}\mathbf{1}H_4]^{2-}$.

Computational modeling of O_2 with inner- and second-sphere water molecules (Supporting Figures S19–S20).

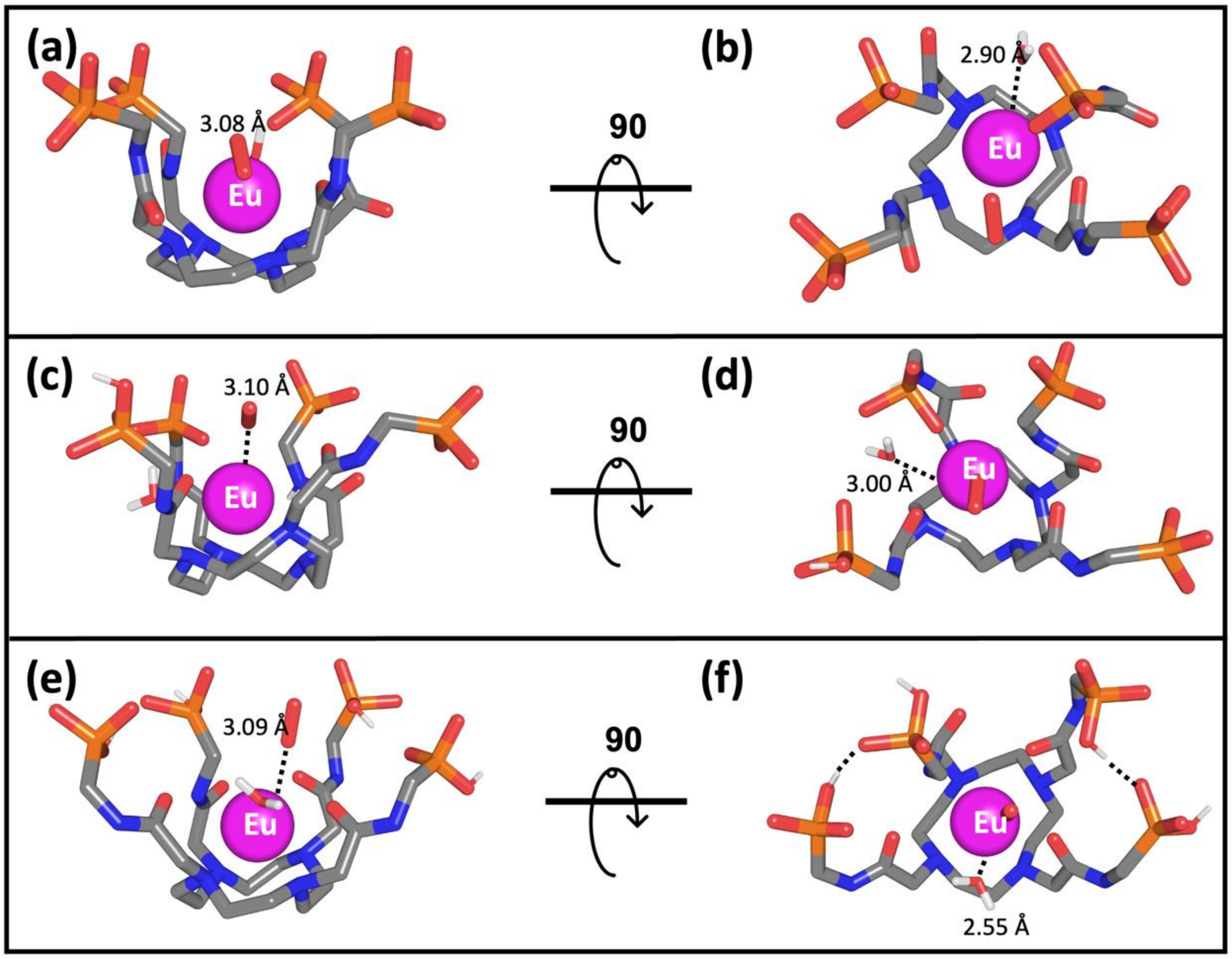

Supporting Figure S19. Representative structures of europium complexes in different protonation states (a) fully deprotonated $[Eu^{II}\mathbf{1}]^{6-}$, (c) mono-protonated $[Eu^{II}\mathbf{1}H]^{5-}$, and (e) tetra-protonated $[Eu^{II}\mathbf{1}H_4]^{2-}$ europium complexes obtained during classical molecular dynamics simulations. The distances to the nearest water molecule and coordinating oxygen atom are indicated.

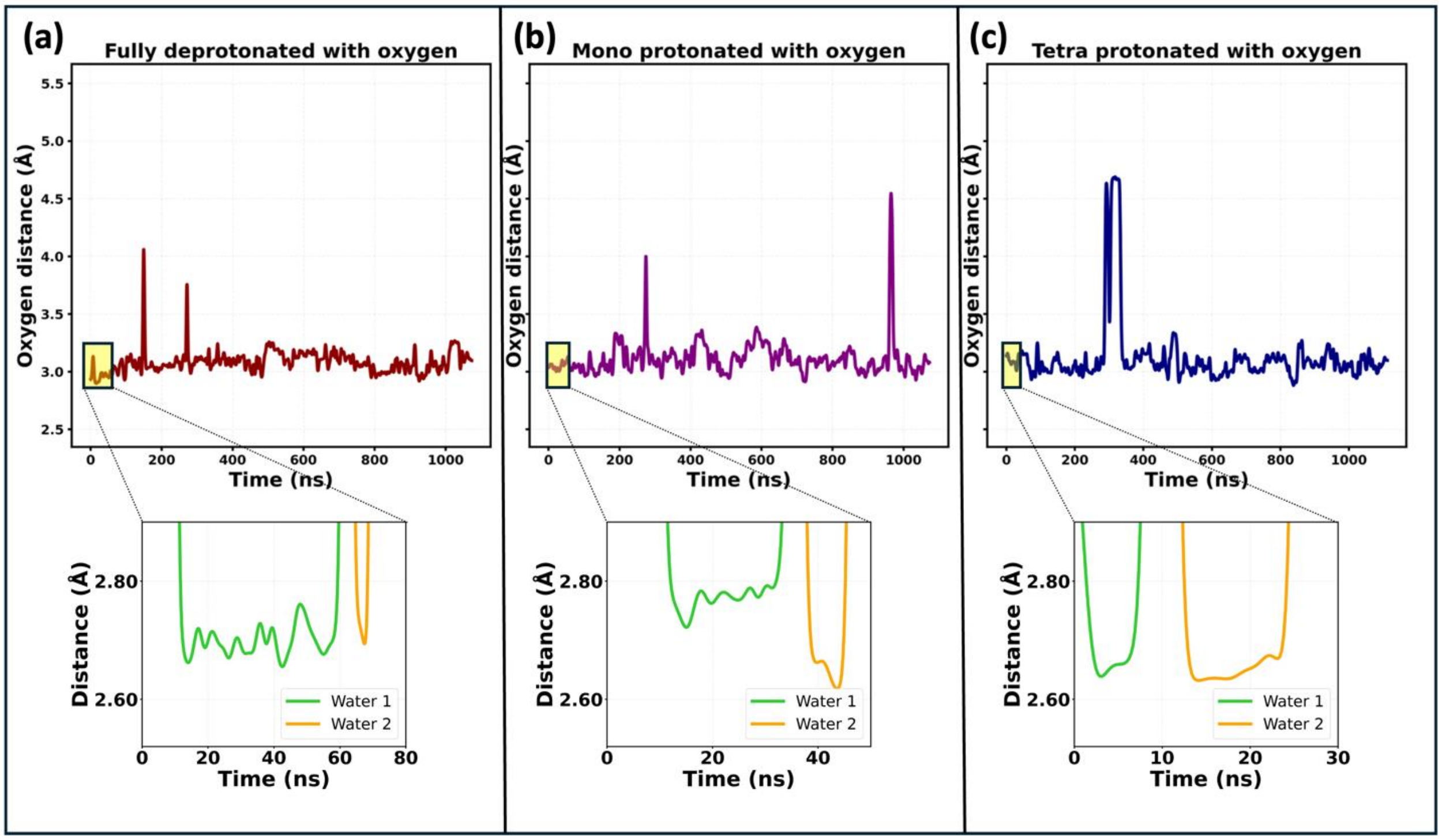

Supporting Figure S20. Eu–O (O_2) distance profiles for (a) fully deprotonated [Eu^{II}**1**]$^{6-}$, (b) mono-protonated [Eu^{II}**1**H]$^{5-}$, and (c) tetra-protonated [Eu^{II}**1**H_4]$^{2-}$ europium complexes obtained during classical molecular dynamics simulations. The highlighted regions in each panel indicate representative water-exchange events. The corresponding subplots provide zoomed-in views of these regions, showing how coordinating water molecules exchange in the presence of the O_2 molecule; similar exchange behavior is observed throughout the simulation.
